# Single-Cell Analysis Reveals Cxcl14^+^ Fibroblast Accumulation in Regenerating Diabetic Wounds Treated by Hydrogel-Delivering Carbon Monoxide

**DOI:** 10.1021/acscentsci.3c01169

**Published:** 2024-01-02

**Authors:** Ya Li, Lu Sun, Ranxi Chen, Wenpeng Ni, Yuyun Liang, Hexu Zhang, Chaoyong He, Bi Shi, Sophie Petropoulos, Cheng Zhao, Liyang Shi

**Affiliations:** ⊥ State Key Laboratory of Chemo/Biosensing and Chemometrics, College of Biology, Hunan University, Changsha 410082, China; | College of Materials Science and Engineering, Hunan University, Changsha 410082, China; § Department of Clinical Science, Intervention and Technology, Division of Obstetrics and Gynecology, Karolinska Institutet, 14186 Stockholm, Sweden; ∥ Département de Médecine, Université de Montréal, Montreal Canada, Centre de Recherche du Centre Hospitalier de l’Université de Montréal, Axe Immunopathologie, H2X 19A 708 Montreal Canada

## Abstract

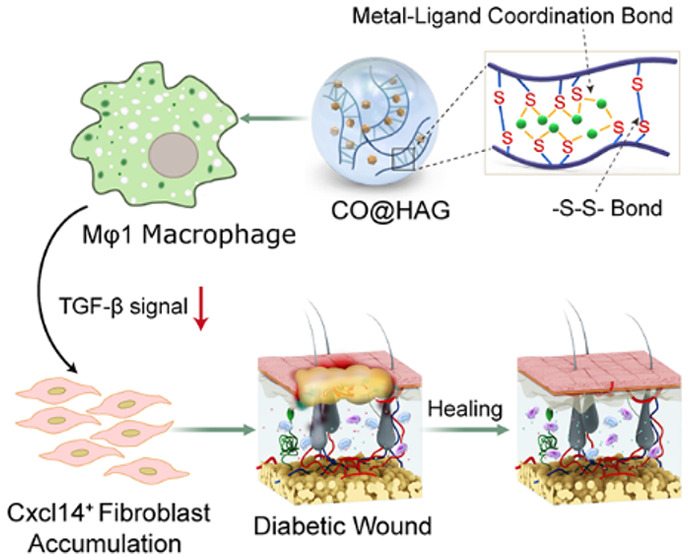

Nonhealing skin wounds are a problematic complication associated with diabetes. Therapeutic gases delivered by biomaterials have demonstrated powerful wound healing capabilities. However, the cellular responses and heterogeneity in the skin regeneration process after gas therapy remain elusive. Here, we display the benefit of the carbon monoxide (CO)-releasing hyaluronan hydrogel (CO@HAG) in promoting diabetic wound healing and investigate the cellular responses through single-cell transcriptomic analysis. The presented CO@HAG demonstrates wound microenvironment responsive gas releasing properties and accelerates the diabetic wound healing process *in vivo*. It is found that a new cluster of *Cxcl14*^*+*^ fibroblasts with progenitor property is accumulated in the CO@HAG-treated wound. This cluster of *Cxcl14*^*+*^ fibroblasts is yet unreported in the skin regeneration process. CO@HAG-treated wound macrophages feature a decrease in pro-inflammatory property, while their anti-inflammatory property increases. Moreover, the TGF-β signal between the pro-inflammatory (M1) macrophage and the *Cxcl14*^*+*^ fibroblast in the CO@HAG-treated wound is attenuated based on cell–cell interaction analysis. Our study provides a useful hydrogel-mediated gas therapy method for diabetic wounds and new insights into cellular events in the skin regeneration process after gas-releasing biomaterials therapy.

## Abstract 2

Carbon monoxide-releasing hyaluronan hydrogel (CO@HAG) promotes Cxcl14^+^ fibroblast accumulation and accelerates diabetic wound healing in a rat model.

## Introduction

Diabetes is a worldwide health crisis that is increasing globally, predicted to reach 600 million by 2035.^1,2^ A major complication in diabetic patients is chronic and nonhealing wounds, such as diabetic foot ulcers, which result in extensive financial and social burdens.^2,3^ Endogenous gaseous signaling molecules were found to participate in many physiological and pathological processes at cell, tissue, and organ levels.^4^ Compared with chemotherapeutic method, administration of exogenous gaseous molecules demonstrated less drug resistance and less cytotoxicity toward normal cells, known as a “green” strategy with negligible side-effects.^5,6^ Recently, gas therapies using biomaterial delivering carbon monoxide (CO),^7,8^ nitric oxide (NO),^9−11^ hydrogen sulfide (H_2_S),^12^ hydrogen (H_2_),^13,14^ oxygen (O_2_)^15,16^ etc. have been applied for treating diabetic wounds. All-in-one CO-releasing versatile chitosan-based hydrogels consumed reactive oxygen species (ROS) and quickly generated CO in tissue defect areas, demonstrating outstanding healing capabilities for diabetic wounds.^8^ Therapeutic NO molecule loading microbubbles were captured into a hydrogel that significantly reduced pro-inflammatory cytokine levels, increased anti-inflammatory cytokine levels, and promoted angiogenesis in diabetic wounds.^10^ A novel polymersome self-assembled from poly(ε-caprolactone)-poly[lysine-*S*-aroylthiooxime] generated H_2_S in the presence of cysteine, promoting diabetic wound areas of epidermal and endothelial cells of proliferation, migration, and angiogenesis.^12^ To use H_2_ molecules of antioxidant and ROS-scavenging abilities, titanium oxide nanorods were prepared to realize visible light-catalyzed H_2_ generation in diabetic wound areas.^14^ O_2_-producing microsphere-based systems accelerated diabetic wound closure because O_2_ promoted keratinocytes and fibroblasts of survival and migration, boosted angiogenic growth factor expression, and reduced pro-inflammatory levels.^15^ Although the above gas-releasing biomaterials demonstrated impressive skin regeneration capacity, the cellular responses and heterogeneity during the healing process are yet unclear.

CO molecules, generally being considered as a silent killer, have strong affinity to hemoglobin (Hb) to form carboxyhemoglobin (COHb). Interestingly, CO can be generated in cells by the known heme oxygenases HO-1 and HO-2. A low concentration of CO acts as a gas signal messenger to regulate several physiological and pathological body processes, such as HO-1 pathway mediated anti-inflammatory reactions.^17−19^ CO of immunomodulatory functionality is also related to the mitogen-activated protein kinase (MAPK) cascade, such as the ERK1/2, JNK, and p38 pathways.^20^ Moreover, CO deactivates toll-like receptor 4 (TLR4) by inhibiting NADPH oxidase activity, thereby downregulating the expression of inflammatory response related genes.^20,21^ In a normal wound healing microenvironment, the subtypes of the macrophage population will transfer easily from predominantly pro-inflammatory (M1) to anti-inflammatory (M2) phenotypes. But diabetic wounds of macrophages continuously remain in the inflammation state, preventing skin from regenerating.^22^ Therefore, using CO to reduce high-inflammatory levels is possible to realize diabetic wound healing. Traditional inhalational delivery is extremely limited for utilization because “uncaged” CO has potential risks to patients and is only suitable for administration in clinics and hospitals with precisely controlled settings.^23^ Applying hydrogels as a CO delivering platform can solve gas therapy of untargetability, easy diffusivity, and short half-time problems. Besides controlling gas therapy, hydrogels can mimic the extracellular matrix (ECM) structure and act as a useful skin substitute.^24,25^

**Figure 1:**
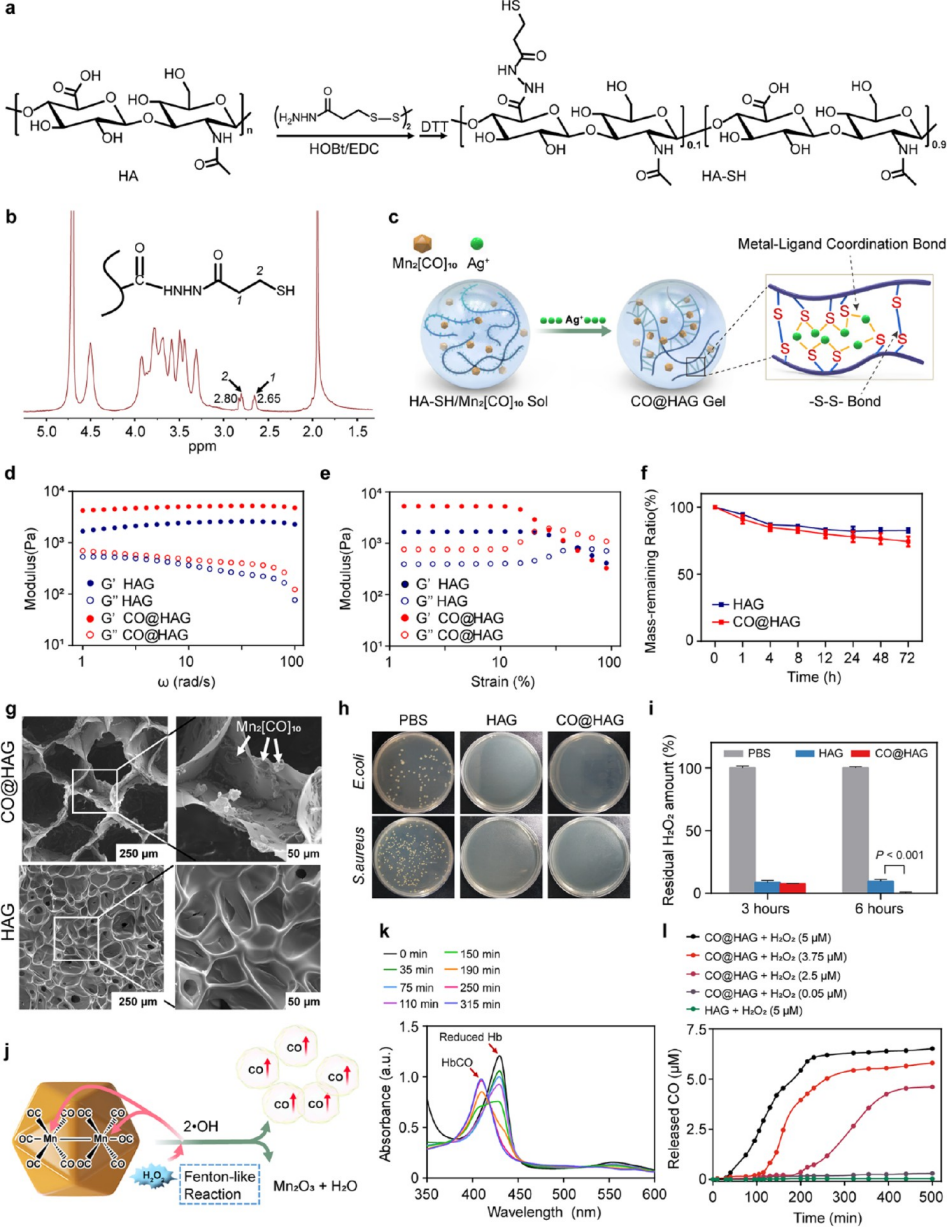
CO@HAG formation and characterization. (a) Schematic of the synthesis of the thiolated hyaluronan (HA-SH) derivative. (b) ^1^H NMR spectrum curve for the HA-SH derivative. (c) Schematic of the CO@HAG formation and the cross-linkages with silver-thiol and disulfide bonds. (d) Storage modulus (*G*′) and loss modulus (*G*″) values of CO@HAG and HAG under a rheological experiment with an angular frequency sweep (1–100 rad/s) at a fixed 0.5% strain. (e) Evolution of *G*′ and *G*′′ values under a rheological experiment with a strain increase from 1% to 100%. (f) Mass-remaining ratio curves of CO@HAG and HAG in 72 h of PBS incubation. (*n* = 3; mean ± s.d.). (g) Microstructure images of lyophilized CO@HAG and HAG using scanning electron microscopy (SEM). (h) Antibacterial experiments of CO@HAG and HAG using the colony-counting method. (i) H_2_O_2_-scavenging results after incubating with CO@HAG and HAG for 3 and 6 h (*n* = 3 to 4; mean ± s.d.). Reported *p* values determined by one-way ANOVA analysis. (j) Schematic of CO release from Mn_2_[CO]_10_ triggered by the H_2_O_2_-mediated Fenton-like reaction. (k) UV–vis spectroscopy monitoring of CO release from CO@HAG in 5 μM H_2_O_2_ solution via the Hb-to-HbCO method. (l) Amount of released CO from hydrogels at different time points in aqueous medium containing various concentrations (0.05, 2.5, 3.75, 5 μM) of H_2_O_2_.

Here, thiolated hyaluronan (HA-SH) derivative is used (Figure 1a,b) with silver ions and a CO-releasing molecule (Mn_2_[CO]_10_) to obtain a CO-releasing hyaluronan hydrogel (CO@HAG). We hypothesize that CO@HAG possesses immunomodulating and healing properties for diabetic wounds. Furthermore, the cellular events in the CO@HAG treated wound microenvironment are investigated using single-cell transcriptomics analysis. After CO@HAG treatment, a new cluster of *Cxcl14*^+^ fibroblast progenitor was accumulated in the wound area, and macrophages’ pro-inflammatory feature was reduced while their anti-inflammatory property was increased. Moreover, TGF-β communication between the M1 macrophage and *Cxcl14*^*+*^ fibroblast was decreased. This study provides a promising CO-based diabetic wound therapy strategy and a deep understanding of dynamic cellular responses and heterogeneity after hydrogel-mediated gas therapy.

## Results and Discussion

### CO@HAG Formations and Characterizations

CO@HAG was fabricated by simply mixing HA-SH/Mn_2_[CO]_10_ sol with Ag^+^ ions, cross-linked by metal–ligand (Ag–S) coordination bonds and disulfide (-S–S-) bonds (Figure 1c). The formation of -S–S- bonds is contributed by oxidation. Moreover, the thiol group in the mixture interacted with Ag(I) to form Ag–S coordination bonds.^26^ HAG as a control hydrogel was prepared from the same concentrations of HA-SH and Ag^+^ ions but without Mn_2_[CO]_10_. Because of dynamic properties of coordination bonds, CO@HAG was molded into desired shapes and two cut pieces of CO@HAG self-healed after joining them together (Figure S1a). The storage modulus (*G*′) values of CO@HAG and HAG were significantly higher than their loss modulus (*G*″) values (Figure 1d). *G*′ was increased from ∼2.5 kPa (HAG) to ∼5.0 kPa (CO@HAG) (Figure 1d), suggesting a more stable network structure after adding Mn_2_[CO]_10_. *G*′ was increased gradually with increasing the Mn_2_[CO]_10_ concentration from 0.5% (w/v) to 1.5% (w/v) (Figure S1b). But the *G*′ value of the hydrogel with 2.0% (w/v) of Mn_2_[CO]_10_ was unexpectedly decreased to ∼3.0 kPa compared with that of the hydrogel with 1.5% (w/v) of Mn_2_[CO]_10_ (*G*′ = ∼ 5.0 kPa), indicating that the polymeric network of integration was interfered with by excess Mn_2_[CO]_10_. The hydrogel was transferred from a gel state into a liquid-like state (*G*′ < *G*′′) when the stain was above 25% in CO@HAG and 50% in HAG (Figure 1e), indicating the hydrogel’s shear-thinning properties. The *G*′ (CO@HAG) value instantaneously recovered to its original value upon shear strain removal, also proving its self-healing character (Figure S1c). After 72 h of incubation, the masses of CO@HAG and HAG (compared with their original mass before swelling) decreased continuously to 74% and 82%, respectively (Figure 1f). Continuous microporous structures in lyophilized CO@HAG and HAG were observed in SEM images (Figure 1g). Compared with CO@HAG, the micropore size and void depth in lyophilized HAG were reduced because of HAG structure collapse during lyophilization. The clinically used Ag^+^ ion (AgNO_3_) concentration is 29 mM, and concentrations of Ag^+^ ions above 60 mM are reported to be toxic to normal tissue.^27,28^ Here, we used 15 mM Ag^+^ ions to prepare CO@HAG, which is four-fold less than the toxic concentration. Moreover, the 15 mM Ag^+^ ion concentration was safely used in our previous paper.^29^ In the CO@HAG hydrogel system, the thiol group of the HA-SH derivative in the aqueous phage is difficult to interact with the solid phage of Mn_2_[CO]_10_ because of steric hindrance. The stability constant for the Ag^+^-organosulfur complex (such as thiol) is around 10^13^,^30^ which is much higher than the constant of 3 × 10^7^ for the Mn-S complex.^31^ Therefore, when Ag^+^ ions mixed with a Mn_2_[CO]_10_/HA-SH mixture, the cross-links of Ag-S were dominantly formed. Moreover, Fourier transform infrared spectroscopy (FT-IR) experiments were used to compare (i) HA-SH+ Mn_2_[CO]_10_ and HA-SH samples and (ii) CO@HAG and HAG samples. After Mn_2_[CO]_10_ incorporation, the resulting difference FT-IR curves revealed the appearance of peaks at 2047 and 2004 cm^–1^ contributed by C≡O vibrations, as well as peaks at 646 and 469 cm^–1^ contributed by Mn–CO vibrations (Figure S2).^32^ The new peak Mn–S was not found in the FT-IR curves, proving no Mn–S bond formation.

Bacterial plaques were not found in colony-counting plates after CO@HAG and HAG treatment, while there were numerous plaques in the PBS group (Figure 1h). According to CCK8 assay, average bacterial inhibition ratios against *E. coli* and *S. aureus* in both the CO@HAG and HAG groups were all >92% (Figure S1d). In diabetic wounds, ROS levels increased by the elevated mitochondrial superoxide products, generation of glycolysis end products, high activity of ROS-formation enzymes, etc.^33^ Imbalance between ROS and antioxidants in wound areas generates cell and tissue damage and inhibits endogenous stem cell proliferation and differentiation. After incubating hydrogels with H_2_O_2_ (a type of nonradical ROS) for 6 h, average residual H_2_O_2_ ratios were <9.5% (Figure 1i). The H_2_O_2_-scavenging properties of CO@HAG and HAG resulted from the oxidation of free thiol moieties and the depletion of H_2_O_2_ by silver. Moreover, in the presence of Mn_2_[CO]_10_, H_2_O_2_ decomposed into ^•^OH catalyzed by the central Mn ion in Mn_2_[CO]_10_ through a Fenton-like reaction. Furthermore, ^•^OH competitively combined with the Mn center and triggered CO release (Figure 1j).^34,35^ The released CO combined with reduced Hb and realized Hb-to-HbCO conversion (Figure 1k and Figure S1e). With the increase of incubation time, the absorbance peak of the reduced Hb gradually decreased and the peak of HbCO constantly increased (Figure 1k). The released CO amount from CO@HAG increased with the increase of H_2_O_2_ concentrations, indicating that CO@HAG has diabetic wound environment responsive CO release properties (Figure 1l and Figure S1f). We used the Hb method for CO detection according to reported references,^34,36−38^ in which H_2_O_2_ is present during the measurement process. The reduced Hb levels of ultraviolet–visible (UV–vis) spectroscopy curves were unchanged after H_2_O_2_ addition, meaning H_2_O_2_ did not influence the reduced Hb structure (Figure S3). Moreover, after adding the HAG hydrogel into the reduced Hb + H_2_O_2_ mixture, the representative peaks did not shift because of no CO release (Figure S3). These results indicated that H_2_O_2_ did not impact the measurement process. Since the Q-band region in the UV–vis curves of HbCO showed low absorbance intensity, the myoglobin (Mb) method was used to detect the Q-band peaks’ conversion according to previous reports.^39−41^ Based on the results in Figure S4a,b, H_2_O_2_ did not impact the absorbance curves of reduced deoxymyoglobin (Deoxy-Mb). HAG cannot induce the conservation of the Deoxy-Mb structure (Figure S4c). Upon adding CO@HAG to Deoxy-Mb solution in the presence of H_2_O_2_, UV–vis curves demonstrated a gradual increase in carbonmonoxy myoglobin (MbCO) formation, which is indicated by two peaks at 541 and 578 nm, along with the decrease of the 555 nm of peak for Deoxy-Mb (Figure S4d). In addition, gas chromatography (GC) results demonstrated that compared with the HAG sample, the typical CO gas peaks at around 6 min appeared in the CO@HAG sample after incubating with H_2_O_2_ (5 μM) medium, further confirming CO release (Figure S5). According to the above results obtained from Hb, Mb, and GC methods, we concluded that the CO is indeed released from CO@HAG.

### Cytocompatibility, Cell Migration and Macrophage Polarization Treated by CO@HAG Precursors

**Figure 2:**
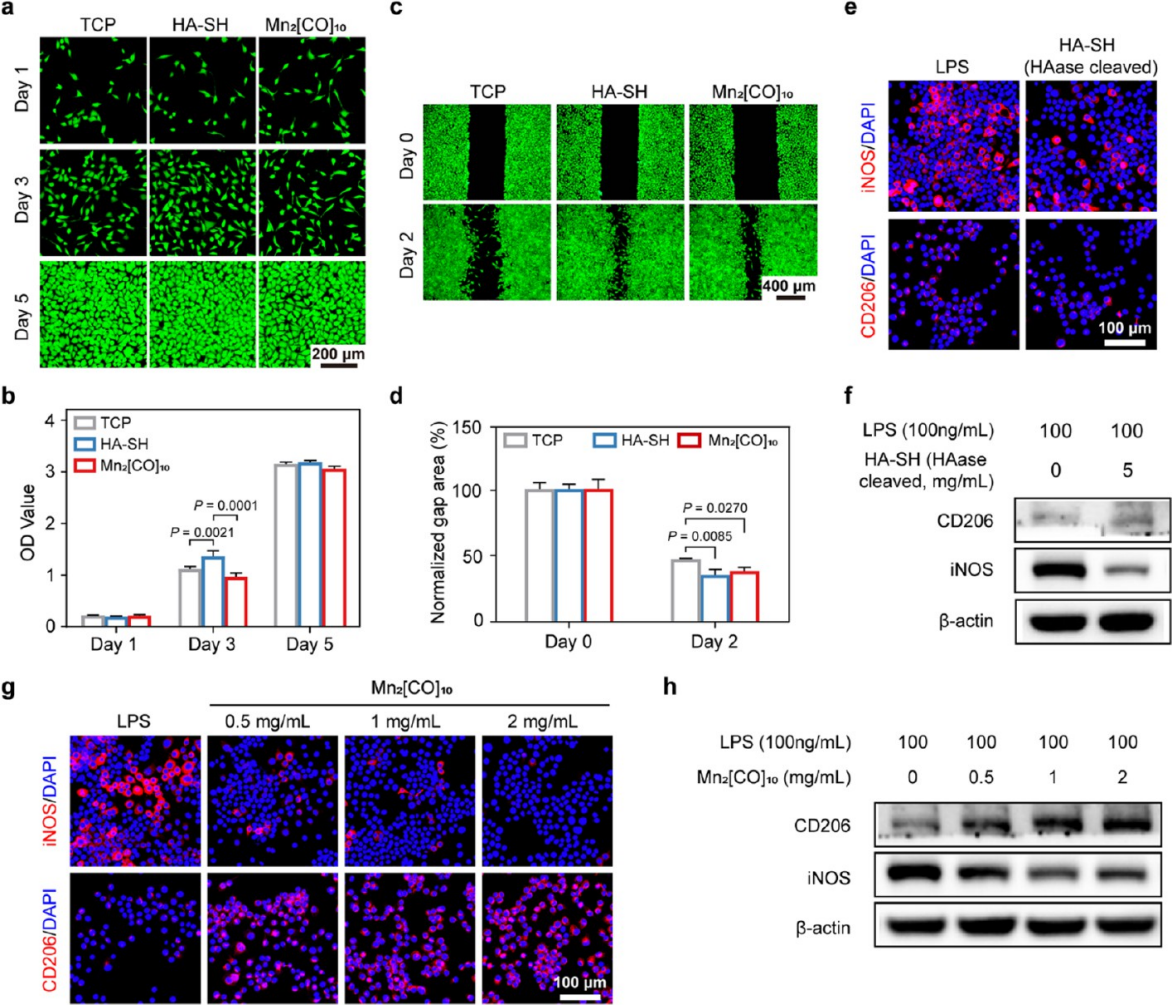
Cytocompatibility, cell migration, and macrophage polarization treated by CO@HAG precursors. (a) Representative fluorescent images of cultured NIH/3T3 cells after 1, 3, and 5 days of incubating with HA-SH and Mn_2_[CO]_10_. Cells cultured on the tissue culture plate (TCP) were used as control. Calcein-AM (green color) was used to stain live cells. (b) CCK8 assay of OD values for cultured NIH/3T3 cells after 1, 3, and 5 days of incubating with HA-SH and Mn_2_[CO]_10_. (*n* = 4; mean ± s.d.). Reported *p* values determined by one-way ANOVA analysis. (c and d) Migration of scratched NIH/3T3 cells in the culture plate without any treatment (TCP) or with addition of HA-SH and Mn_2_[CO]_10_. (*n* = 3; mean ± s.e.m.). Reported *p* values determined by one-way ANOVA analysis. (e) Representative immunofluorescent images of LPS-induced macrophage treated by HA-SH for 24 h (red, iNOS or CD206; blue, cell nuclear). (f) Western blotting (WB) measuring macrophages’ iNOS and CD206 protein levels after HA-SH treatment. (g) Representative immunofluorescent images of LPS-induced macrophage treated by Mn_2_[CO]_10_ for 24 h (red, iNOS or CD206; blue, cell nuclear). (h) WB measuring macrophages’ iNOS and CD206 protein levels after Mn_2_[CO]_10_ treatment.

Cytocompatibility is one of the important properties for skin regenerative biomaterial. Considering fibroblasts play an important role in the wound healing process,^42^ NIH/3T3 fibroblast cells (isolated from mouse embryo) were mainly used to evaluate biomaterials’ cytocompatibility. NIH/3T3 cells grew with the increase of culturing time in two CO@HAG precursor-treated groups (HA-SH and Mn_2_[CO]_10_) (Figure 2a,b), suggesting that they were nontoxic to NIH/3T3 cells. As shown in Figure 2c,d, HA-SH and Mn_2_[CO]_10_ significantly accelerated the NIH/3T3 cells’ migration compared with the cells culturing on the tissue culture plate (TCP) without HA-SH and Mn_2_[CO]_10_ addition.

Macrophages play an important role in normal wound healing by eliminating cell debris and coordinating defect repair, and macrophages in diabetic wounds are characterized by a persistent pro-inflammatory state.^43,44^ Therefore, using biomaterials to transfer macrophages from pro-inflammatory M1 type to M2 type can effectively promote the healing process. Hyaluronidase (HAase)-cleaved HA-SH effectively decreased lipopolysaccharides (LPS)-induced RAW 264.7 cells (a macrophage cell line) of M1-type marker (iNOS) expression, while it did not influence the M2-type marker (CD206) (Figure 2e,f). On the other hand, Mn_2_[CO]_10_ molecules not only decreased LPS-induced macrophages of iNOS expression but also increased CD206 expression (Figure 2g,h). The intensities for the decreased iNOS level and increased CD206 level depended on the Mn_2_[CO]_10_ concentration (Figure 2h). The cytometry results showed that the inhibition rates for HA-SH and Mn_2_[CO]_10_ against iNOS-positive cells were ∼40% and ∼65%, respectively (Figure S6). The above data suggested that both HA-SH and Mn_2_[CO]_10_ can inhibit M1 macrophage polarization, but only Mn_2_[CO]_10_ can switch the M1 macrophage into M2-type. To confirm that the biological effects of Mn_2_[CO]_10_ are due to CO molecules, we prepared a post-release metal fragment (i.e., inactive Mn_2_[CO]_10_ (i-Mn_2_[CO]_10_)) that was produced by leaving Mn_2_[CO]_10_ in PBS buffer containing H_2_O_2_ at room temperature for 48 h to allow all CO to be liberated from the molecule.^45,46^ Based on immunofluorescence staining and WB assay, compared with the LPS treated group, the intensities of pro-inflammatory (iNOS) and anti-inflammatory (CD206) markers were unchanged in the i-Mn_2_[CO]_10_ group, showing that i-Mn_2_[CO]_10_ is of limited immunomodulating properties (Figure S7a,b). NIH/3T3 cell experiments demonstrated that i-Mn_2_[CO]_10_ cannot promote fibroblast migration (Figure S7c,d). Together with the results in Figure 2, the biological effects of CO@HAG are mainly caused by CO instead of the Mn complex.

### CO@HAG Accelerated Diabetic Wound Healing and Regulated Wound Cell Heterogeneity

**Figure 3:**
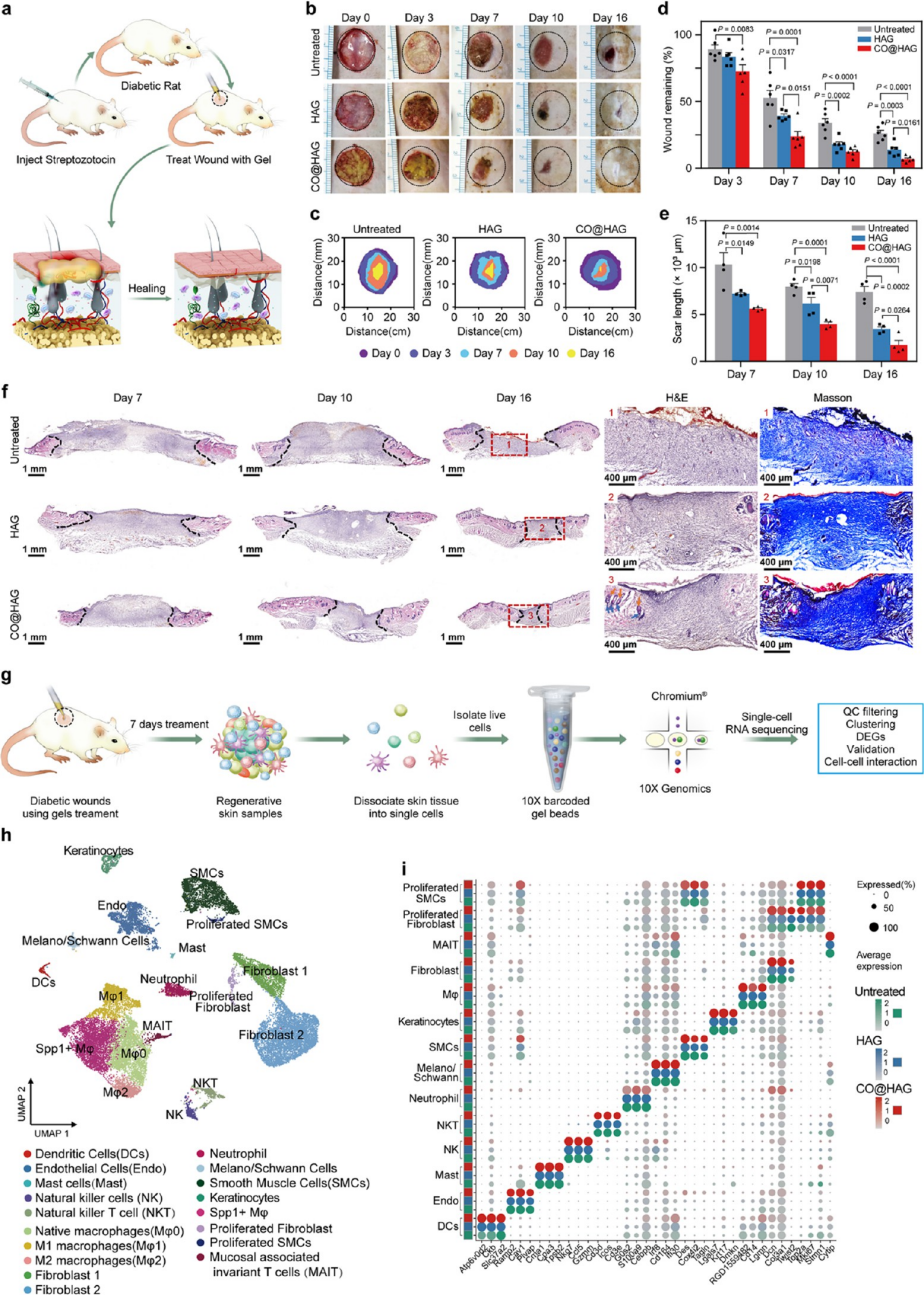
CO@HAG accelerated diabetic wound healing and regulated wound cell heterogeneity. (a) Schematic of the construction of the diabetic wound and the wound healing process after hydrogel therapy. (b) Wound sizes were monitored by camera at post CO@HAG and HAG treatment of 0, 3, 7, 10, and 16 days. The same volume of PBS with hydrogel was used as the untreated group. (c) Schematic of wound area traces at post hydrogel treatments of 0, 3, 7, 10, and 16 days. (d) Wound remaining rates at post hydrogel treatments of 3, 7, 10, and 16 days. Wound remaining rates were calculated by comparing wound sizes at different time points with the original wound size at 0 day (*n* = 6; mean ± s.e.m.). Reported *p* values determined by one-way ANOVA analysis. (e) Scar length at post hydrogel treatments of 7, 10, and 16 days were measured based on the H&E staining sections. (*n* = 4; mean ± s.e.m.). Reported *p* values determined by one-way ANOVA analysis. (f) Images of wound microstructures based on H&E and Masson’s staining. Blue arrows represent hair follicles, and orange arrows represent sebaceous glands. (g) Schematic of the single-cell analysis process. (h) Uniform Manifold Approximation and Projection (UMAP) plot of all cells from the untreated, HAG, and CO@HAG samples, colored by cell type. (i) Dot plots showing candidate marker genes specific to each cell type. Dot size and color encode the percentage of cells and average expression level within each group (green, blue, and red represent high expression).

The skin regeneration ability of CO@HAG was evaluated in a diabetic rat full-thickness skin defect model (Figure 3a). Based on images and schematic of wound area traces, CO@HAG accelerated the diabetic wound healing process compared with HAG and the untreated (PBS) group (Figure 3b,c). Noticeable wound area was not found in the CO@HAG group after 16 days of treatment. From 3 to 16 days, the average wound remaining rates gradually decreased from 72.57 ± 4.89% to 6.71 ± 0.92% in the CO@HAG group, from 83.58 ± 2.98% to 13.77 ± 1.97% in the HAG group, and from 89.05 ± 3.38% to 26.00 ± 2.33% in the untreated group, respectively (Figure 3d). Wound scar lengths in the above three groups were gradually reduced (Figure 3e,f). After 16 days of treatment, the average wound scar lengths of the HAG and untreated groups were 3458.13 and 7400.70 μm, respectively, while that of the CO@HAG group significantly decreased to only 1745.90 μm. Hair follicles (blue arrows) and sebaceous glands (orange arrows) were noticeably found in the regenerative skin area after treatment with CO@HAG. But the typical morphology of skin appendages was not found in the HAG and untreated groups (Figure 3f). Collagen, a key component in the ECM, plays an important role in the regulation of the wound healing process by either its native, fibrillar, or soluble component, which has been used as a key evaluation parameter for the wound healing efficacy.^47^ The collagen deposited density in the CO@HAG group was higher than that in the HAG and untreated groups, and the collagen fibers were longer (Figure 3f). Therefore, the fabricated CO@HAG can effectively accelerate the diabetic wound healing process.

To comprehensively examine the range of cell types and transcriptomes involved in wound healing, we isolated cells samples from wounds after treating with CO@HAG, HAG, and PBS (untreated) and generated single-cell transcriptome libraries using the droplet-based 10X Genomics Chromium system (Figure 3g). Cells from the untreated, HAG, and CO@HAG samples finally resulted in a total of 24,880 sequenced cells that met quality control metrics. The cell clusters obtained from UMAP embedding analysis revealed 18 distinct cell types (Figure 3h and Figure S8a). To accurately classify the cell types, we examined the differentially expressed gene signatures of each cluster and cross-referenced them with known markers (Figure 3i and Table S1). The most abundant cluster, accounting for 38.5% of all cells, was identified as macrophages, which highly expressed the myeloid markers *RGD1559482* and *Cd14* (Figure S8b and Table S1). The second most abundant cluster was identified as fibroblast cells, which were enriched with *Dcn* and *Pdgfra* (Figure 3i and Figure S8b).^48,49^ Smooth muscle cells (SMCs) were classified based on the expression of *Des* and *Tagln*,^48^ while endothelial cells (Endo) were enriched with *Cav1* and *Ramp2*, Melano/Schwann cells with *Irf8*, Mast cells (Mast) with *Cpa3*, Dendritic cells (DCs) with *Ckb*, and Neutrophil cells with *G0s2* and *S100a9*. Additionally, two clusters belonging to fibroblast and SMCs were identified as undergoing proliferation based on the expression of the proliferating markers *Top2a* and *Mki67*.^50^ Furthermore, we analyzed the proportion of each cell type in each sample (Figure S8c), although limited by statistical power (N = 1 biological replicate). Our findings suggested that CO@HAG treatment caused an alteration in the proportion of macrophages (from 44.4% in the untreated group to 25.3% in the CO@HAG group) and fibroblast cells (from 24.6% in the untreated group to 44.8% in the CO@HAG group), which suggests that CO@HAG treatment has the effects of decreasing the inflammatory environment and promoting fibroblast proliferation.

### CO@HAG Inhibited Wound Macrophages of Pro-inflammatory but Promoted Anti-inflammatory Features

**Figure 4:**
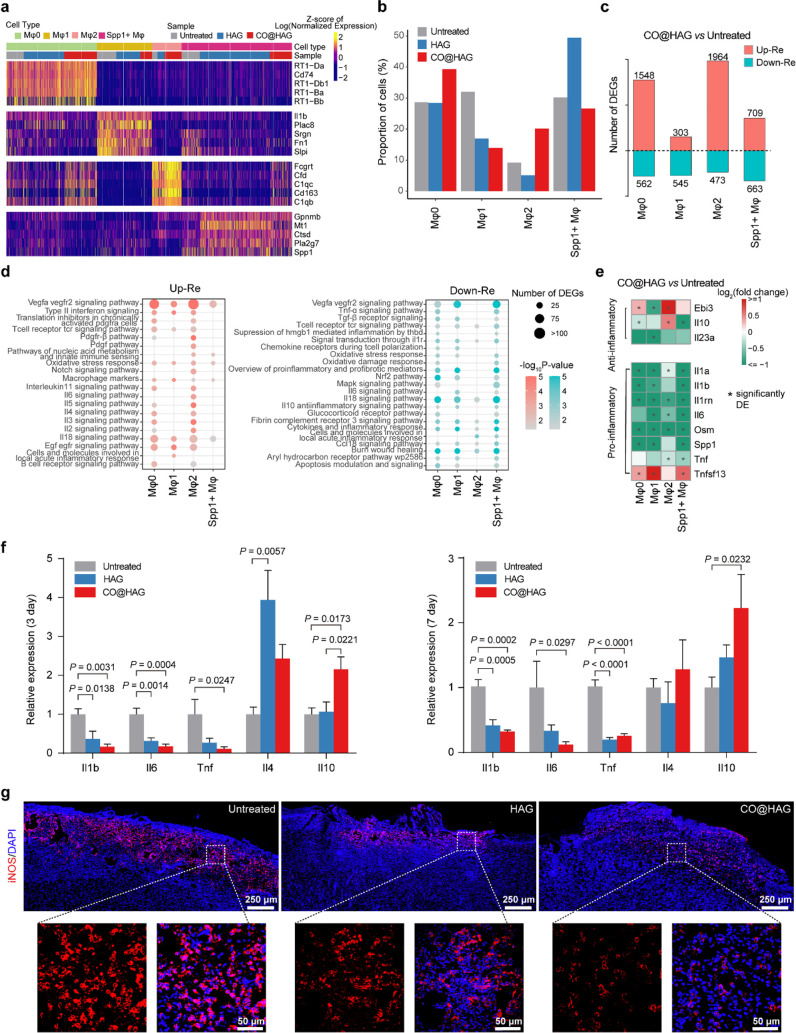
CO@HAG inhibited wound macrophages of pro-inflammatory but promoted anti-inflammatory features. (a) Heatmap of marker gene expression for the 4 subclusters of macrophages based on single-cell transcriptome data. Marker genes were selected based on “power” (roc test), with the top 5 selected if there were more than 5 markers. (b) Bar plots showing the proportion of different macrophage subclusters in the untreated, HAG, and CO@HAG samples (gray, blue, and red, respectively). (c) The number of differentially expressed genes (DEGs) between the untreated and CO@HAG samples in macrophage subcluster cells. Numbers above and below the *x*-axis represent the number of up- and downregulated DEGs in the CO@HAG samples, respectively. (d) Pathway enrichment analysis of significantly differentially expressed genes following CO@HAG treatment. Circle size and color represent the number of DEGs and pathway significance, respectively. (e) Heatmap showing the log-transformed fold change between CO@HAG-treated and untreated samples for known anti-inflammatory and pro-inflammatory genes based on single-cell transcriptome data. Significantly differentially expressed genes are labeled with “*”. (f) RT-qPCR results of relative expression levels of pro-inflammatory and anti-inflammatory genes in skin samples after 3 and 7 days of application with untreated, HAG, and CO@HAG. (*n* = 3 to 4; mean ± s.e.m.). Reported *p* values determined by one-way ANOVA analysis. (g) Immunofluorescence images stained with iNOS and DAPI for skin tissues obtained from 7 days of untreated, HAG, and CO@HAG therapy.

Based on the enriched expression of *Cd74*, *Il1b*, *Cd163*, and *Spp1*, we identified M0, M1, M2, and *Spp1*^*+*^ macrophage cells using Figure 4a.^48,51^ We observed that CO@HAG treatment had opposite effects on the proportions of M1 macrophage and M2 macrophage, suggesting its potential to inhibit pro-inflammation and promote anti-inflammatory effects (Figure 4b). Interestingly, the HAG sample displayed less differentiation of the M1 macrophage and was efficiently polarized into the *Spp1*^*+*^ macrophage instead of the M2 macrophage, which might be attributed to the shortage of CO releasing or some other factors. To assess the impact of CO@HAG treatment on the transcriptome of each macrophage subtype, we performed differential expression analysis between CO@HAG-treated and untreated samples within each subtype (Figure 4c, Figure S9a, and Table S2). We identified a total of 2,808 upregulated and 1,329 downregulated genes between the CO@HAG and the untreated samples (Figure S9a and Table S2). Among the significant differentially expressed genes (DEGs) between the CO@HAG and untreated groups, 82 and 95 genes were upregulated and downregulated in all subtypes of macrophages, respectively (Figure S9a and Table S2). The overlapped upregulated genes included *Ly6e* (Lymphocyte antigen 6 complex locus E) (Figure S9b), which has been suggested to play important roles in immunological regulation, particularly in the regulation of inflammatory responses as well as in the development and activation of T cells.^52^ Pathway enrichment analysis revealed that genes related to the Pdgf pathway, Pdgf-beta pathway, and IL2, IL3, IL4, IL5, and IL6 signaling pathways were upregulated in M2 macrophages, while genes related to the TGF-β receptor signaling pathway were significantly downregulated in all types of macrophages except M2 macrophages (Figure 4d). Compared with the untreated group, most pro-inflammatory genes, like *Il1a* (interleukin 1 alpha), *Il1b* (interleukin 1 beta), and *Il6* (interleukin 6), were significantly downregulated after CO@HAG therapy in macrophage clusters. On the other hand, anti-inflammatory genes including *Il10* (interleukin 10) were exclusively upregulated in the M2 macrophage (Figure 4e). Moreover, genes related to “overview of pro-inflammatory and profibrotic mediators” were downregulated in the M0, M1, and *Spp1*^*+*^ macrophages but not in the M2 macrophage (Figure S10). Genes related to the Vegfa-vegfr2 signaling pathway exhibit a more complex expression pattern in macrophages. Both up- and downregulated DEGs related to this pathway were found to be enriched in the M0, M1, and *Spp1*^*+*^ macrophages, while only upregulated DEGs were enriched in the M2 macrophages. The Vegfa-vegfr2 axis is closely associated with angiogenesis and is also expressed in the macrophage lineage cells, stimulating inflammatory responses in various tissues.^53^ A previous study reported that the Vegfa-vegfr2 axis boosted THP1 (human monocytic cell line)-derived macrophage migration and improved M2 phenotype macrophages’ polarization.^54^

Pro-inflammatory genes (*Il1b, Il6, Tnf*) and anti-inflammatory genes (*Il4, Il10*) of mRNA levels from the 3- and 7-days treated wound samples were detected by RT-qPCR (Figure 4f). Compared with the untreated group, the mRNA levels of *Il1b*, *Il6*, and *Tnf* in both the HAG and CO@HAG groups were significantly downregulated at 3 and 7 days. Compared with the untreated group, CO@HAG treatment upregulated the mRNA levels of *Il10* at 3 and 7 days; however, HAG did not affect the *Il10* levels (Figure 4f). Moreover, tissue immunofluorescence images showed that the M1 macrophage marker (iNOS) was evidently reduced in the CO@HAG group compared with the untreated group (Figure 4g). Overall, these above RT-qPCR and tissue immunofluorescence results corresponded with the single-cell analysis of results that CO@HAG therapy can reduce the inflammatory responses of diabetic wounds. Only 10.5% and 12.0% of DEGs in the CO@HAG samples were also differentially expressed in HAG samples when compared to untreated samples in the M0 and M2 macrophages, respectively (Figure S9c,d). In contrast, DEGs in the M1 and *Spp1^+^* macrophages were more commonly regulated in both the HAG and CO@HAG samples, with 41.9% and 58.1% of DEGs being expressed in both sample types, respectively (Figure S9c,d). Interestingly, the genes related to the Vegfa-vegfr2 signaling pathway we previously mentioned were uniquely upregulated DEGs in CO@HAG for the M0 and M2 macrophages, which may be explained by the anti-inflammatory effects that were more related to CO release (Figure S9e). This observation aligns with the *in vitro* results in Figure 2e–h that the CO-releasing molecules but not the HA-SH polymer promoted anti-inflammatory features. Furthermore, Gene Ontology (GO) enrichment analysis was conducted for genes exclusively differentially expressed in CO@HAG samples (Figure S11). The analysis revealed upregulation in GO terms such as “intracellular protein transport”, “protein folding”, “Golgi vesicle transport”, and “protein transport” in the M0 and M2 macrophages of CO@HAG samples. Those fundamental biology process are vital for the proper functioning of macrophages. Additionally, GO terms related to the “I-kappaB kinase/NF-kappaB cascade” and “negative regulation of the I-kappaB kinase/NF-kappaB cascade” were enriched in the uniquely downregulated DEGs of the M0 and Spp^+^ macrophages. The I-kappaB kinase (IKK)/NF-kappaB cascade, a critical signaling pathway in cells, regulates various biological processes, including immune responses, inflammation, cell proliferation, differentiation, and survival.^55^ Those results confirmed the importance of CO-releasing.

### Cxcl14^+^ Fibroblasts Function as Progenitors in Regenerating Wound

**Figure 5:**
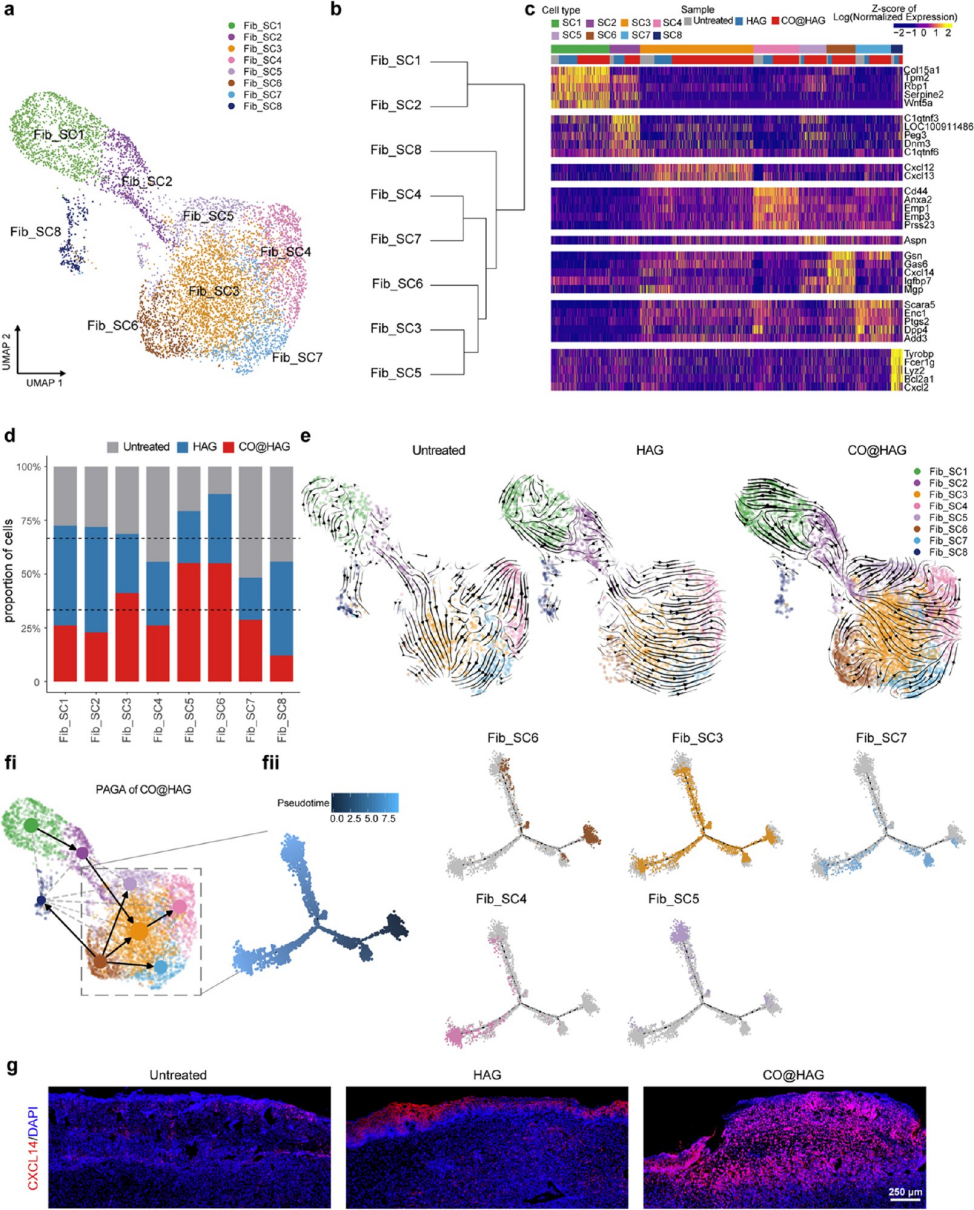
Fibroblast heterogeneity and *Cxcl14*^*+*^ fibroblast function as a regenerative progenitor in diabetic wounds. (a) Subclustering of wound fibroblasts (cells from Fibroblast1 and Fibroblast2 in Figure 3) identified 8 distinct subtypes. A color-coded UMAP plot is shown, and each fibroblast subcluster (SC1 through SC8) is defined on the right. (b) Unsupervised hierarchical clustering showing the relatedness of wound fibroblast subclusters. (c) Heatmap showing the expression of marker genes for the 8 subclusters of fibroblasts. Marker genes were selected using the “power” (“roc” test), and the top 5 were chosen if they had more than 5 markers. (d) Barplot showing the relative proportion of cells from untreated, HAG, and CO@HAG samples for each fibroblast subcluster. (e) RNA velocities of each sample projected onto the UMAP space as in part a. (fi) Partition-based graph abstraction (PAGA) analysis of the CO@HAG fibroblasts based on RNA velocity information. (fii) Trajectory inference color-coded by inferred pseudotime and cell types for dermal fibroblast subclusters in the CO@HAG sample. (g) Skin immunofluorescence images demonstrating CXCL14 protein level differences in untreated, HAG, and CO@HAG samples.

Considering the crucial role of fibroblasts in the wound healing process, including their ability to produce and organize ECM components, provide structural support, and facilitate tissue repair, we conducted a detailed analysis of fibroblast cells. We performed unsupervised clustering on fibroblast cells only by subsetting and integration analysis (Figure 5a, see Experimental Section). By requiring at least one highly expressed marker (power > 0.4, see Experimental Section), we further divided 7,669 fibroblast cells (excluding proliferating fibroblast cells) into 8 subclusters: Fib_SC1 through Fib_SC8 (Figure 5b). Among them, Fib_SC1 and Fib_SC2 (25.3% in total) belonged to the previous Fibroblast1 cluster and behaved dramatically differently from the rest of the clusters (previous Fibroblast2 cluster). Hierarchical clustering on the spearman correlation among different clusters, calculated based on all expressed genes, also confirmed the close and far relationships among those clusters. We found that the marker genes *Mafb* and *Gpc1* were highly expressed in Fib_SC1 and Fib_SC2. In contrast, *Plac8*, *Dpt* and *Sfrp4*, *Tgfbr3* had the opposite expression pattern in other fibroblast clusters (Figure S12 and Table S1). *Mafb*(56) and *Gpc1*(57) were found to be localized in the skin epidermis layer. *Sfrp4*,^58^*Plac8*,^59,60^*Dpt*,^61^ and *Tgfbr3*,^62^ were reported to be highly expressed in the dermis or deep region of the dermis layer. We postulate that these two major fibroblast populations represent the two types of fibroblasts from the epidermis and dermal layers, respectively. Fib_SC1 may represent the *Cd24*^*+*^ fibroblast reported previously,^51^ and Fib_SC2 with high expression of *C1qtnf3* may represent the paramysial fibroblast^49^ (Figure 5c, Figure S12, and Table S1). Fib_SC3 with high expression of *Cxcl12*, Fib_SC7 with high coexpression of *Dpp4* and *Pi16*, and Fib_SC5 with high expression of *Aspn* have been reported in the perturbed-state mouse fibroblast atlas.^50^ Fib_SC4 with high expression of *Prss23* could be related to systemic sclerosis driven by fibroblast-differentiated myofibroblasts.^63^ Additionally, Fib_SC8, which highly expressed *Lyz2*, may represent the type of myofibroblasts with hematopoietic features, which also matches the spatial information that it is likely generated from the dermal fibroblast.^62^ Fib_SC6 highly expresses *Gsn*, *Gas6*, *Cxcl14*, and *Mgp*, especially *Cxcl14* that was exclusively highly expressed in Fib_SC6, indicating that it could be a novel fibroblast cluster that was yet unreported in the skin regeneration process.

To gain a deeper understanding of the roles played by subtypes of fibroblasts during wound healing, we observed an increased proportion of Fib_SC 5 and 6 in the CO@HAG sample compared to other samples (Figure 5d). This may indicate that these subtypes play important roles in wound healing. To test this hypothesis, we further investigated the dynamic nature of fibroblast heterogeneity based on the expression patterns of unspliced and spliced mRNAs (RNA velocity).^64^ Our analysis supported an interesting shifting of differentiation trajectories in fibroblasts. We observed one directional stream from epidermal Fib_SC2 in the untreated sample to other dermal fibroblasts, while in contrast, two streams are likely generated from not only epidermal Fib_SC2 but also dermal Fib_SC6 to other dermal fibroblasts in CO@HAG samples (Figure 5e). Partition-based graph abstraction (PAGA) also confirmed the second differentiation stream, including one originating from Fib_SC6 to others in the CO@HAG samples (Figure 5fi). Developmental trajectory ordered by pseudotime also supports the possibility that Fib_SC6 could function as a root cluster or potential source of regenerative progenitors among dermal layer fibroblasts (Figure 5fii). From this, we observed one minor bifurcation to generate Fib_SC7 and one major bifurcation to generate Fib_Sub4 and Fib_Sub5 during dermal layer fibroblast differentiation. Additionally, Fib_SC3 worked as a transition type of fibroblast instead. Moreover, skin tissue immunofluorescence images revealed that the CXCL14 protein expression in the CO@HAG sample was higher than that in the HAG and untreated samples (Figure 5g). It was reported previously that CXCL14 is expressed by many types of nonimmune skin cells such as dermal fibroblasts, keratinocytes, dermal endothelial cells, and so on.^65^ A previous study reported that CXCL14-overexpressing fibroblasts promoted fibroblasts’ migration and proliferation in a prostate tumor model because of autocrine CXCL14-stimulation.^66^ We examined the differential gene expression between CO@HAG samples and untreated samples in Fib_SC6 (Figure S13) and identified a total of 252 upregulated and 286 downregulated DEGs. Among these DEGs, several well-known genes associated with cell differentiation, growth, and migration were upregulated in CO@HAG samples (Figure S13a). For instance, “Egr1” (Early growth response protein 1), a transcription factor, and “Mapk3” (Mitogen-activated protein kinase 3), a kinase in the MAPK signaling pathway, were upregulated; they play roles in regulating cell growth, differentiation, and responses to extracellular signals.^67,68^ Similarly, “Tcf7l2” (a transcription factor involved in the Wnt signaling pathway) and “Ripk1” (Receptor-interacting serine/threonine-protein kinase 1), involved in cell survival, inflammation, and programmed cell death, were upregulated.^69,70^ In contrast, genes such as “Sfrp1” (Secreted frizzled-related protein 1), acting as a Wnt antagonist, and “Birc3” (Baculoviral IAP repeat-containing protein 3), an inhibitor of apoptosis protein (IAP) involved in apoptosis regulation and cell survival, were downregulated in CO@HAG-treated Fib_SC6 cells (Figure S13a). Further GO enrichment analysis validated the upregulation of genes related to “positive regulation of Wnt protein secretion” and downregulation of genes associated with “positive regulation of apoptotic cell clearance” (Figure S13b). It was previously reported that Wnt signaling is activated during wound healing and deeply participated in each of the steps of the healing process, from arranging inflammation regulation, to stem cell mobilization, to matrix remodeling.^71,72^ Together with these published references and our results, therefore, we believe that *Cxcl14*^*+*^ fibroblasts function as regenerative progenitors during wound healing.

### Receptor–Ligand Analyses Revealed Differential Interactions between the M1 Macrophage and Cxcl14^+^ Fibroblast

**Figure 6:**
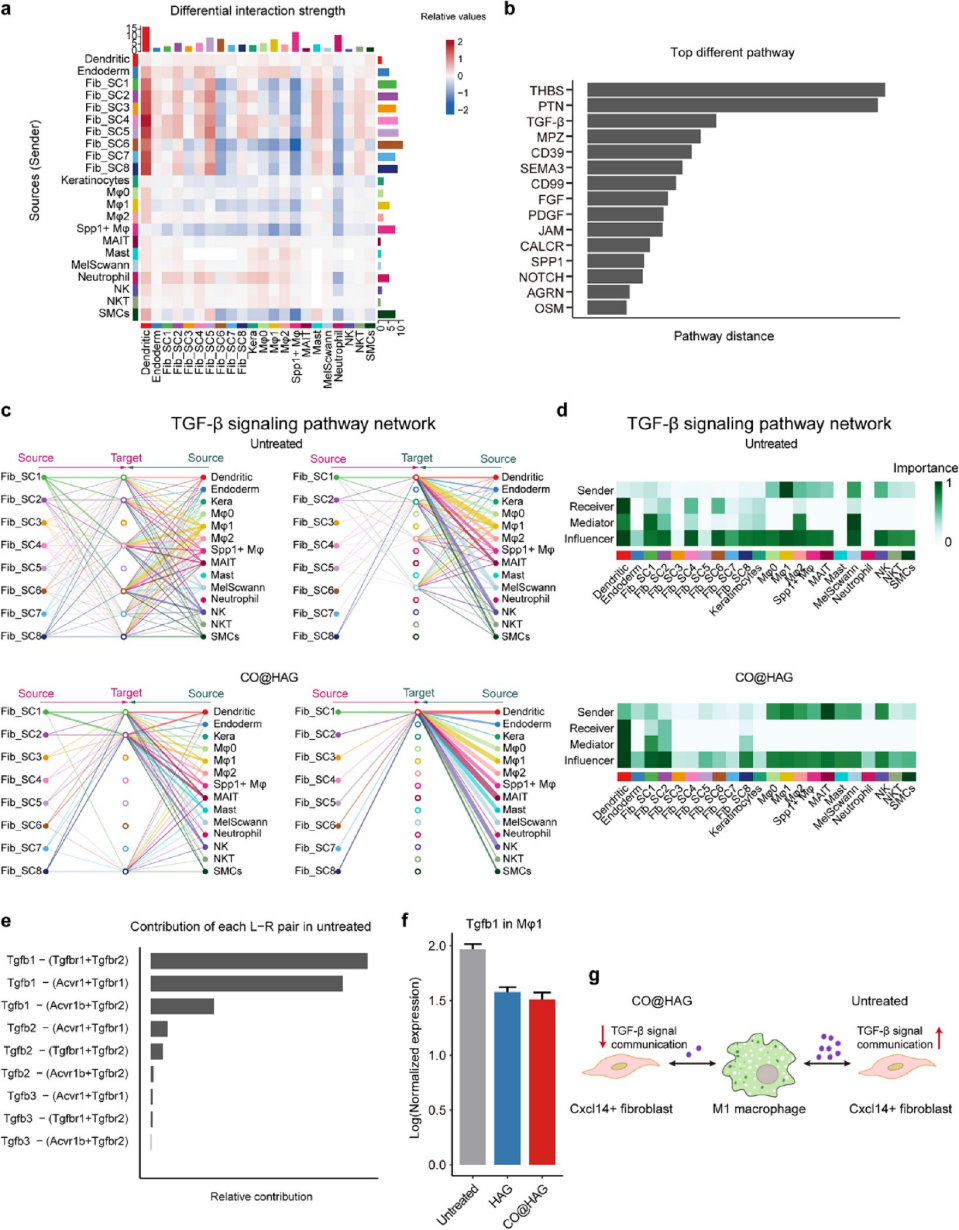
Joint identification of conserved and context-specific communication patterns between untreated and CO@HAG samples. (a) Heatmaps showing the differential strength of interactions between the untreated and CO@HAG samples, displaying the outgoing and incoming signaling changes of each cell group. The top-colored bar plot represents the sum of each column of values displayed in the heatmap (incoming signaling), while the right-colored bar plot represents the sum of each row of values (outgoing signaling). In the color bar legend, red or blue represents increased or decreased signaling in the CO@HAG sample compared with the untreated sample, respectively. (b) Barplot showing the Euclidean distance of signaling networks between the untreated and CO@HAG samples in the shared two-dimensional space. The top 15 signaling pathways are shown here. (c) Hierarchical plot showing fibroblast cells and nonfibroblast cell interactions via TGF-β signaling in the untreated and CO@HAG samples. The left and right portions display the autocrine and paracrine signaling to fibroblast cells and nonfibroblast cells, respectively. Circle sizes are proportional to the number of cells in each cell group, and edge width represents the communication probability. (d) Computed network centrality measures of TGF-β signaling. (e) Barplot showing the contribution of each ligand–receptor pair to the TGF-β signaling pathway in the untreated sample. (f) Barplot showing the log-transformed normalized expression of Tgfb1 in Mφ1. The error bar in the graphs represents the standard error of the mean. (g) Schematic demonstrating the attenuated TGF-β communication between M1 macrophages and Cxcl14^+^ fibroblasts in the regenerating wound area.

Since cell–cell interactions always influenced cellular behaviors and fates, the potential interactions of the presented fibroblasts with other major cell types in the skin were investigated. We utilized the cell–cell interaction analysis by querying the receptor–ligand database^73^ and observed substantial differences in receptor–ligand pairing possibilities between the CO@HAG and untreated samples (Figure 6a). Among the top three differentially regulated signaling pathways were the thrombospondin (THBS), pleiotrophin (PTN), and TGF-β signaling pathways (Figure 6b). The TGF-β signaling pathway is a complex and influential network involving wound fibroblasts, with multiple ligands identified as driving fibroblast–fibroblast, fibroblast–myeloid, and fibroblast–endothelial interactions.^74^ Therefore, we further investigated cell–cell interactions specific to the TGF-β signaling pathway. In the untreated sample, Fib_SC1, 2, 4, 6, 7, and 8 were the main subtypes receiving ligands belonging to the TGF-β signaling pathway (Figure 6c). The network centrality analysis of the TGF-β signaling pathway is shown in Figure 6d. The M1 macrophage was the dominant sender and influencer of TGF-β to dendritic cells, Fib_SC2, Fib_SC4, and Fib_SC6. Additionally, Fib_SC1 and Fib_SC2 acted as mediators, while Fib_SC7 and Fib_SC8 mainly functioned as influencers (Figure 6d). In contrast, the number of receptor–ligand pairing possibilities of fibroblasts with other cell types was limited in the CO@HAG samples, with Fib_SC4 and Fib_SC6 losing their receiver roles (Figure 6c,d). The dominance of Tgfb1 receptor–ligand pairing in the TGF-β signaling pathway could explain the relief out of the TGF-β signal pathway, which could be related to decreased expression of Tgfb1 in the HAG and CO@HAG samples compared with the untreated sample (Figure 6e,f). This also explains why we observed a similar decrease in TGF-β communication in the HAG samples (Figure S14).

Based on the above results, we proposed that the attenuated TGF-β communication between M1 macrophages and *Cxcl14*^*+*^ fibroblasts after CO@HAG therapy promotes *Cxcl14*^*+*^ fibroblast accumulation at the wound site, further leading to an accelerated skin regeneration process (Figure 6g). Although decreased TGF-β communication was also observed in the HAG treatment, the shortage of CO compared to CO@HAG resulted in reduced anti-inflammatory effects (Figure 4 and Figure S9) that could impair the benefits of progenitor fibroblast accumulation. TGF-β including three isoforms TGF-β1, TGF-β2, and TGF-β3 is a family of pluripotent cytokines, and TGF-β1 plays a dominant role in cutaneous wound healing.^75^ It is reported that the TGF-β1 signal was involved in several stages of wound healing and had both positive and negative effects on the healing process.^76^ Although many animal models exhibited decreased TGF-β1 expression in impaired wound healing, transgenic mice overexpressing TGF-β1 in keratinocytes demonstrated delayed wound re-epithelialization in the full-thickness defect model.^77^ The high TGF-β1 level at the wound microenvironment impaired the wound healing, which may be partially attributed to its pro-inflammatory effect.^76^ Our result that the M1 macrophage-*Cxcl14*^*+*^ fibroblast of TGF-β communication was reduced in healed wounds corresponded with these reported results, providing a new understanding of TGF-β signaling in the skin regeneration process.

## Conclusions

In summary, we designed and fabricated a hyaluronan-based hydrogel with CO-releasing behavior (CO@HAG) that possessed antibacterial, anti-inflammatory, antioxidation, and wound microenvironment responsive gas releasing properties. CO@HAG with self-healing, shear-thinning, and moldability characteristics benefited the process of filling and painting irregular-shaped wounds in the clinic. Compared with the untreated sample and the hydrogel without CO (HAG), CO@HAG significantly *in vivo* accelerated the diabetic wound healing process, decreased the scar length, and promoted collagen deposition. Based on single-cell transcriptomics analysis, an unreported cluster of progenitor fibroblasts with overexpressing *Cxcl14*^+^ gene was found to be accumulated in the CO@HAG-treated wound. Cell-chat mediated cellular receptor–ligand analysis demonstrated that the TGF-β communication between the M1 macrophage and *Cxcl14*^*+*^ fibroblast was attenuated after CO@HAG therapy. Overall, this study provides a new gas therapeutic strategy for nonhealing diabetic wounds and a novel insight into the cellular responses and heterogeneity in skin regeneration after gas therapy.

## Supporting Information Available

Experimental Section including HA-SH derivative preparation, fabrications and characterizations of CO@HAG and HAG, antibacterial and H_2_O_2_-scavenging experiments, CO gas release measurement, cytocompatibility assay and cellular migration study, macrophage polarization experiments, diabetic wound healing study, immunofluorescence staining, real-time qPCR analysis, etc. Single-cell analysis experiments including cell capture, scRNA-Seq library preparation, reads mapping and gene expression quantification, data quality control and normalization, data integration, dimensionality reduction, clustering, marker gene detection and differential gene expression analysis, RNA velocity, trajectory interference pseudotime analysis, cell–cell interactions analysis, etc. Supplementary figures include CO@HAG formations and characterizations, flow cytometry analysis results, CO@HAG regulated regenerating wound of cellular heterogeneity, CO@HAG and HAG differentially regulated wound macrophage of inflammatory responses, cell–cell interactions in the HAG group, etc. (PDF) (PDF)
List marker genes associated with the main cell types and subtypes for fibroblasts and macrophages (Table S1) (XLS)
Identify differentially expressed genes between CO@HAG-treated and untreated samples, as well as HAG-treated and untreated samples within each cell type (Table S2) (XLS)
Gene primer sequences (Table S3) (XLSX)
