# Supplementary material for: Single-Cell Analysis Reveals Cxcl14+ Fibroblast Accumulation in Regenerating Diabetic Wounds Treated by Hydrogel-Delivering Carbon Monoxide
Source: ACS Cent Sci. 2024 Jan 2;10(1):184–98. doi: 10.1021/acscentsci.3c01169 (PMC10823591; doi:10.1021/acscentsci.3c01169)
Supplement: Supplementary file 1 — oc3c01169_si_001.pdf [file oc3c01169_si_001.pdf]

# Supporting Information

**Single-Cell Analysis Reveals Cxcl14$^{+}$ Fibroblast Accumulation in Regenerating Diabetic Wound Treated by Hydrogel-Delivering Carbon Monoxide**

Ya Li†‡, Lu Sun†‡, Ranxi Chen†, Wenpeng Ni丨, Yuyun Liang†, Hexu Zhang†, Chaoyong He†, Bi Shi†, Sophie Petropoulos§‖, Cheng Zhao§*, Liyang Shi†*

†State Key Laboratory of Chemo/Biosensing and Chemometrics, College of Biology, Hunan University, Changsha, 410082, China

丨College of Materials Science and Engineering, Hunan University, Changsha 410082，China

§Department of Clinical Science, Intervention and Technology, Division of Obstetrics and Gynecology, Karolinska Institutet, 14186 Stockholm, Sweden

‖ Département de Médecine, Université de Montréal, Montreal Canada; Centre de Recherche du Centre, Hospitalier de l'Université de Montréal Axe Immunopathologie, H2X 19A Montreal, Canada

* Corresponding authors. Liyang Shi (E-mail): liysh777@hnu.edu.cn; Cheng Zhao (E-mail): cheng.zhao@ki.se

‡Y.L. and L.S. contributed equally to this work.

## Experimental Section

### Materials preparation

Hyaluronan (HA) (200~400 kDa) was purchased from Bloomage Biotechnology Co. Ltd. (Jinan, China). DL-dithiothreitol (DTT), 1-ethyl-3-(3-dimethylaminopropyl) carbodiimide (EDC), N- hydroxy benzotriazole (HOBt), and acetonitrile were purchased from Aladdin Co. Ltd. (Shanghai, China). Sodium hydrosulfite, manganese carbonyl ($Mn_2[CO]_{10}$) and 4', 6-diamidino-2-phenylindole (DAPI) were purchased from Sigma-Aldrich Co. Ltd. (USA). Bovine hemoglobin, hematoxylin, eosin, and masson's trichrome stain kit were purchased from Beijing Solarbio Science & Technology Co. Ltd. (Beijing, China). Hyaluronidase (HAase) was obtained from Shanghai Yuanye Bio-Technology Co., Ltd. $H_2O_2$ quantitative assay kit (Water-Compatible) and agar were purchased from Sangon Biotech Co. Ltd. (Shanghai, China). Yeast extract and tryptone were purchased from BioDee Co. Ltd. (Beijing, China). Cell Counting Kit-8 (CCK8) reagent was purchased from Bestbio. (Shanghai, China). Streptozotocin was purchased from Meilun Biotech. (Dalian, China). Silver nitrate was purchased from Tianjin Kemiou Chemical Reagent Co. Ltd. (Tianjin, China). PBS buffer and paraformaldehyde fixative (4%) were purchased from Servicebio Service BioTechnology Co. Ltd. (Wuhan, China). Optimum Cutting Temperature (OCT) compound was purchased from Applygen Technologies Inc. (Beijing, China). Above commercial reagents and solvents were used as received without further purification. RAW264.7 cell, NIH3T3 cell and cell culture medium were purchased from Procell Life Science and Technology Co, Ltd (Wuhan, China). 3,3'-Dithiobis (propionic hydrazide) was synthesized according to the previous literature.[1] Preparation of HAase cleaved HA-SH solution: 3 mL of 5 mg/mL HA-SH solution was mixed with 10 μL of 100 mg/mL HAase solution, and the mixture was incubated at 50 °C for 45 min to cleave HA-SH. Subsequently, the HAase-cleaved HA-SH solution was incubated at 70 °C for 1 h to inactivate HAase.

### Synthesis of HA-SH derivative

400 mg of HA (corresponding to 1 mmol carboxyl group) were dissolved with 60 mL $H_2O$ followed by addition of 16.7 mg (0.07 mmol/L) of 3'3-Dithiobis(propionic hydrazide), 135 mg of HoBt (1 mmol) in 3 mL of 1:1(V/V) acetonitrile: deionized water, 57.5 mg of EDC (0.3 mmol). The above mixture was reacted at room temperature overnight under pH 4.5-5 environment, thereafter 54.5 mg of DTT (0.25 mmol) was added into the reaction mixture to cleave disulfide bond and obtain HA-SH. The structure of resulting derivative after dialysis and lyophilization were measured with $^1H$-NMR spectra (400 MHz, $D_2O$). The new appearance of peaks at 2.80 and 2.65 ppm in the $^1H$-NMR curves of HA-SH derivative were attributed by protons of methylene group (-C$\underline{H_2}$C$\underline{H_2}$SH) side chain on HA backbones (Figure 1b). The thiol group modification density in HA-SH polymer was around 10%, calculated by the integration of the above methylene protons compared with the acetamide group of the acetyl-glucosamine unit in the HA backbone.

**Fabrications and characterizations of CO@HAG and HAG**

CO@HAG was formed by simply mixing HA-SH sol (2% (w/v)), $Mn_2[CO]_{10}$ dispersion (1.5% (w/v)), and $AgNO_3$ solution (15 mM). Particularly, to prepare 1 mL of CO@HAG hydrogel, firstly 20mg HA-SH was dissolved into 700 μL $H_2O$ and 15 mg $Mn_2[CO]_{10}$ was dispersed in 200 μL $H_2O$. Thereafter HA-SH solution and $Mn_2[CO]_{10}$ dispersion were quickly mixed and 100 μL of 150 mM of $AgNO_3$ solution was immediately added into the above mixture under vortexing. The hydrogel without $Mn_2[CO]_{10}$ was represented as HAG. Clinical used $Ag^+$ ion ($AgNO_3$) solution of concentration is 29 mM and above 60 mM of $Ag^+$ ions are reported to be toxic to normal tissue.[2, 3] The 15 mM $Ag^+$ ions inside the presented CO@HAG is four-ford less than the toxic concentration, and the same concentration was also safely used in our previous paper.[4] Rheological properties of CO@HAG and HAG were investigated using MCR-92 advanced rheometer (Anton Paar, Austria) with a 15-mm-diameter parallel plate. Storage modulus (G') and loss modulus (G'') of hydrogels containing different $Mn_2[CO]_{10}$ concentrations were measured by frequency sweep experiments (from 1 to 100 rad/s) at a fixed strain (0.5%). Moreover, CO@HAG and HAG of G' and G'' were

monitored under the strain's gradual increase from 1% to 100% at a fixed 1 Hz frequency. SEM (TESCAN, Czech Republic) was used to observe the microstructure of the lyophilized CO@HAG and HAG. Mass-remaining ratio curves for CO@HAG and HAG were investigated in PBS medium with pH 7.4, and the mass-remaining ratio (%) (n = 3) was calculated by dividing hydrogel mass after each time point of incubating to the initial mass before incubation.

**Hydrogels anti-bacterial and $H_2O_2$-scavenging experiments**

*E.coli* (ATCC 25922, Gram-negative strains) and *S.aureus* (ATCC 25923, Gram-positive strains) were used to evaluate the anti-bacterial properties of CO@HAG and HAG. 1 mL of bacterial solution with $10^7$ CFU/mL was co-cultured with 200 μL of CO@HAG and HAG on a 24-well plate for 12 hours under 37℃. 200 μL of PBS instead of hydrogel as control group was cultured with the above bacterial solutions. CO@HAG, HAG and PBS-treated bacterial solutions were diluted with the same dilution ratio and further cultured on Luria-Bertani (LB) agar medium for 12 hours. Thereafter the remaining colonies were observed using optical photographs. To quantify hydrogels-treated remaining bacterial viabilities, the OD values at 450 nm were recorded by SpectraMax M2 instrument (Molecular Devices, US) according to CCK8 assay. We investigated hydrogels of non-radical ROS ($H_2O_2$) scavenging abilities using the $Fe^{2+}$- $Fe^{3+}$ transition of Fenton reaction method. Particularly, CO@HAG and HAG were incubated with 1 mL 100 μM $H_2O_2$ solution and the residual $H_2O_2$ amounts after 3 and 6 hours of incubating were measured using a $H_2O_2$ quantitative assay kit (Sangon Biotech, Shanghai).

**CO gas release measurement**

The released CO amount was measured using the hemoglobin (Hb)-carboxyhemoglobin (HbCO) conversion method. Particularly, 4.2 μM of bovine hemoglobin was dissolved in PBS buffer and reduced with 1.6 mg of sodium dithionite (SDT) under a nitrogen protection environment. 200 μL of CO@HAG was incubated with 4 mL reduced Hb solution containing 5 μM of $H_2O_2$. Immediately, the above

reaction mixture was sealed in a UV quartz cuvette and the UV absorbances were recorded from 350 nm to 600 nm using a UV-Vis spectrophotometer (UV-6100A, Metash Instruments Co.Ltd., China) at the certain time-points. 200 μL of HAG under 5 μM $H_2O_2$ medium was used as negative control. Moreover, the UV absorbance at 410 and 430 nm for the CO@HAG under different $H_2O_2$ concentrations of medium (0.05, 2.5, 3.75, 5 μM) were recorded to calculate the CO releasing amount using the Figure S1f equation.[5] $I_{410nm}$ and $I_{430nm}$ represent the OD value of the collected spectrum at 410 nm and 430 nm, respectively. $C_{CO}$ and $C_{Hb}$ represent releasing CO amount and the initial Hb amount (4.2 μM), respectively.

**Cytocompatibility assay and cellular migration study**

NIH3T3 cells (CRL-1658, ATCC) were used to evaluate the cytocompatibility of HA-SH and $Mn_2[CO]_{10}$. Particularly, NIH3T3 cells ($1.5 \times 10^3$ cells/well) were seeded in 96-well plates and incubated in DMEM complete medium with 10% (v/v) Fetal Bovine Serum (FBS) and 1% (v/v) Penicillin-Streptomycin. After overnight, culture mediums were replaced by complete mediums containing HA-SH solution (2 mg/mL) or $Mn_2[CO]_{10}$ dispersion (2 mg/mL). After 1, 3, and 5 days culturing, the absorbances at 450 nm for cell viabilities was recorded using the CCK8 assay. NIH3T3 cells were stained by Calcein/PI Live/Dead Assay Kit (Beyotime, C2015M) to visualize the cellular growth, and the stained cells were imaged with inverted fluorescence microscope (Nikon, Japan). For cellular migration experiment, NIH3T3 cells ($5 \times 10^5$ cells/well) were seeded in 6-well plates. After cell layer was formed on plate, a straight scratch in NIH3T3 cell lay was made by a 200 μL pipette tip to mimic wound environment. The culture medium with 1% (v/v) FBS and 1% (v/v) Penicillin-Streptomycin containing HA-SH solution (2 mg/mL) or $Mn_2[CO]_{10}$ dispersion (2 mg/mL) were used to culture the scratched cells for 48h. The migration area was observed with a fluorescence microscope after staining living cells with Calcein-AM dye. The migration ratios were calculated by normalizing against the gap area at 0h in Image J software.

**LPS-induced macrophage of polarization experiments**

RAW264.7 cells (TIB-71, ATCC) ($3 \times 10^4$ cells/well) were seeded in 48-well plates for overnight. Thereafter, the cells were incubated with HA-SH solution (HAase cleaved) (5 mg/mL) and $Mn_2[CO]_{10}$ (0.5, 1 and 2 mg/mL) with the presence of LPS (Sigma, 100 ng/mL) for 24 hours. RAW264.7 cells were fixed with paraformaldehyde (4%, v/v, Labcoms Life Sciences) for 15 min, permeabilized with Triton X-100 (0.1%, w/v, Beyotime) for 15 min, followed by being blocked with FBS (5%, v/v, Gibco) for 1 hour. The treated cells were incubated with the anti-iNOS antibody (1:400, abcam, ab178945) or anti-Mannose Receptor antibody (1:500, abcam, ab64693) overnight at 4 °C and secondary antibody solution (Alexa Fluor 546-conjugated Goat anti-Rabbit Secondary Antibody (1:500, Invitrogen, A-11035)) for 1 hour, then stained with DAPI dye for 15 min. The fluorescence images were visualized on confocal laser scanning microscopy (CLSM, Nikon, Japan). As for WB assay, the supernatants of treated cells were obtained with passive lysis buffer (Promega, E1941) containing cocktail (Sangon, C600387) and phenylmethylsulfonyl fluoride (PMSF, Beyotime, P0100). The protein was separated by SDS-PAGE and transferred to the polyvinylidene fluoride (PVDF) membrane. After blocking with skim milk (5%, w/v) for 1 hour, the PVDF membrane was incubated with the Anti-iNOS antibody (1:1000), anti-Mannose Receptor antibody (1:1000) and β-actin (1:1000, Proteintech, 20536-1-AP) overnight at 4 °C, respectively. The membranes furtherly were incubated with HRP-conjugated Goat anti-Rabbit IgG (H+L) (Abiowell, AWS0002a, 1:10000) for 1 hour. The targeted protein bands were visualized on Automatic Gel Imaging Analysis System with the presence of enhanced chemiluminescence (ECL) reagents (Thermo Scientific, 34580). As for cytometry measurements, the treated cells were also analyzed using flow cytometry (CytoFLEX, Beckman, USA) after incubating with the PE anti-Nos2 (iNOS) Antibody (1:500, Biolegend, 696806) overnight.

**In vivo diabetic wound healing study**

SD male rats (6 - 8 weeks of age; Hunan Sta Laboratory Animal Co., Ltd., China) were intraperitoneally injected streptozotocin (65 mg/kg). The rats with blood glucose over

16.7 mM were used to prepare skin full-thickness defects with 15 mm of diameter under anesthesia. The prepared 300 μL of CO@HAG or HAG was painted onto each wound area and the wounds were protected with sterilized gauze. The same volume of PBS was used as untreated group. Digital images of the wound area were captured with photo camera at post-therapy of 0, 3, 7, 10 and 16 days, and the wound closure rate was analyzed using Image J software. Wound tissues were obtained and embedded in the OCT compound after fixation with 4% paraformaldehyde. For the histological analysis, the 10 μm of frozen slices were stained with Haematoxylin & Eosin (H&E) and Masson's Staining Kit. Images of stained slices were captured using a Digital Slice Scanning System (Pannoramic MIDI, 3DHISTECH, Hungary). All experimental procedures involving animals were approved by the Institutional Animal Care and Use Committee of College of Biology, Hunan University (Approval NO. HNUBIO202101006).

**Skin immunofluorescence staining**

All sections were washed three times with PBS and permeabilized in 0.1% TritonX-100 for 15 min at room temperature, followed by being blocked with 5% FBS for 1 hour at room temperature. The blocked sections were incubated with primary antibody at 4°C overnight. After washing three times with PBS, all sections were incubated with corresponding secondary antibodies for 1 hour and DAPI dye for 15 min at room temperature. The images of skin tissue sections were captured with an Ultra-high resolution confocal microscope (LSM980, ZEISS, Germany). Herein used primary antibodies included anti-iNOS antibody (ab178945, 1:400) and anti-CXCL14 antibody (ab264467, 1:100). Secondary antibody (from Invitrogen) used in the study was Alexa Fluor 546-conjugated Goat anti-Rabbit Secondary Antibody (A-11035, 1:1000).

**Skin real-time qPCR analysis**

Harvested skin tissues were washed with PBS and total RNA was extracted with Trizol (Solarbio, China) according to the protocols. The concentration of extracted total RNA was determined using Nano Drop spectrophotometer (Nanodrop2000, Thermo Fisher

Scientific). Complementary DNA (cDNA) was synthesized by reverse transcription of the total RNA (1 μg) using reverse transcriptase (Bimake, USA). The relative mRNA expression levels (*Il1b, Il6, Tnf, Il4,* and *Il10*) were quantified using Hieff® qPCR SYBR Green Master Mix (Yeasen, China) by real-time fluorescence quantitative PCR instrument (CFX96, Bio-rad, USA). Data were analyzed using the $2^{-\Delta\Delta Ct}$ method. The cycle threshold (Ct) values of target gene were normalized using the Ct values of *Gapdh* gene. Primer sequences were listed in Table S3.

**Single-cell capture and scRNA-Seq library preparation**

Fresh wound skin samples were obtained from 7 days of untreated, HAG and CO@HAG groups, cleaned with PBS, and digested using the Skin Dissociation Kit (Miltenyi biotec, 130-101-540) for ~1 hours at 37℃. Then the filter was used to purify the above cell suspension, and ACK buffer (Lonza, 10-548E) was used to remove red blood cell. The resulting cells were necessary to reach >90% of cell viabilities and resuspended in PBS containing 0.04% Bovine Serum Albumin (BSA). The cells were immediately captured by Single Cell 3' Library and Gel Bead Kit V3.1(10x Genomics, 1000121) and Chromium Single Cell G Chip Kit (10x Genomics, 1000120). The cell suspension with 300-600 living cells/μL was loaded onto the Chromium single cell controller (10x Genomics) to generate single-cell gel beads in the emulsion. The captured cells were lysed inside gel bead-in-emulsions (GEMs) and the released RNA was barcoded through reverse transcription using S1000TM Touch Thermal Cycler (Bio Rad) at 53℃ for 45 min, followed by 85℃ for 5 min, and hold at 4℃. The generated cDNA was amplified, and quality estimated using Agilent 4200 (performed by CapitalBio Technology, Beijing). The sequences of libraries were finally obtained using the Illumina Novaseq6000 sequencer with a sequencing depth of 25,000~40,000 reads per cell and pair-end 150 bp (PE150).

**Reads mapping and gene expression quantification**

Single-cell cDNA library samples were sequenced as described above. Demultiplexing, alignment, and quantification of single-cell fastq files were performed using the Cell

Ranger Pipeline v7.0.0 (10x Genomics)[6] with default settings against the *Rattus norvegicus* reference genome (v.6.0, release 104, downloaded from the Ensemblwebsite).[7]

**Data quality control and normalization**

Low-quality cells were removed based on the number of expressed genes (nGene) and the expression level of mitochondrial genes (percent.mito). Specifically, cells with percent.mito less than 0.15 and 750 < nGene < 6000 were retained. Doublet discrimination was implemented using the doubletFinder_v3 function in the DoubletFinder package (v.2.0.3) for each sample individually.[8] Following the selection of high-quality cells, we further filtered based on genes. Genes that excluded mitochondrial genes were considered truly expressed if they contained one or more counts in at least five cells (assessed for each sample separately). Log-normalized counts were calculated using the deconvolution strategy implemented by the computeSumFactors function in the scran package (v.1.14.6).[9] Rescaled normalization was performed using the multiBatchNorm function in the batchelor package (v.1.2.4) to ensure that the size factors were comparable across samples.[10] The log-normalized expression after rescaling was used in marker gene detection and differential gene expression analysis.

**Data integration, dimensionality reduction, and clustering**

We integrated the filtered count matrices from 3 samples using the sctransform approach implemented in the Seurat package (v.4.2.0) on the 3000 anchor features.[11] After integration, principal component analysis (PCA) was performed on the integrated data followed by embedding into low-dimensional space with Uniform Manifold Approximation and Projection (UMAP) based on the top 40 dimensions. Clusters were generated by graph-based method using the FindClusters function from the Seurat package and assigned to cell types by consulting the expression of known marker genes and automatic annotation from the SingleR package (v.1.4.1).[12] Proliferating cells were identified by the high-level expression of *Top2a,* and *Stmn1* and excluded from further

analysis. To identify detailed types of fibroblast cells, cells from the 2 big fibroblast clusters were further extracted and integrated based on 2000 anchor features and top 30 dimensions. Sub-fibroblast clusters were further identified using the FindClusters function based on fibroblast-only PCA dimensions and by requiring at least one highly expressed marker (power > 0.4 using ‘roc’ test) (Figure 5; Figure S12, Supporting Information). Raw counts for cells belonging to the same fibroblast subclusters were aggregated into a pseudo-bulk sample. Normalization of expression for pseudo-bulk samples was calculated using the estimateSizeFactors function, followed by the estimateDispersions function from the R package DESeq(v.1.38.0).[13] The log-transformed and normalized gene expression values of expressed genes from pseudo-bulk data were used to calculate the Spearman correlation among different subclusters, followed by hierarchical clustering by the hclust function in R.

**Marker gene detection and differential gene expression analysis**

Marker genes for each cell type were identified using the FindAllMarkers function with the "roc" test from the Seurat package. The top 50 marker genes with an average power of at least 0.4 were selected (Table S1). Differential gene expression analysis among samples in the macrophage clusters was performed using the "MAST" test implemented in the FindMarkers function from the Seurat package.[14] Genes with an FDR less than 0.05, log2(fold change) greater than 0.25, and expressed in more than 15% of the cells were considered differentially expressed. The scaled gene expression in the untreated, HAG, and CO@HAG samples were checked by the radial ggplot function in the volcano3D package.[15] The gene ontology (GO) enrichment analysis for differentially expressed genes was conducted using the TopGO package (v.2.42.0).[16] Adrian Alexi's improved weighted scoring algorithm and Fisher's test were used to define the significance of GO term enrichment. Functional enrichment analysis and gene set enrichment analysis (GSEA) were performed using the "enricher" and "GSEA" function from the clusterProfiler package(v.3.18.1),[17] respectively. Wiki pathway annotations and gene sets for GSEA analysis were downloaded from the Molecular Signatures Database (MSigDB) database.[18-20] Significantly enriched GO and functional

terms were identified as those with a *p*-value and FDR less than 0.05, respectively.

**RNA velocity, trajectory interference, and pseudotime analysis**

RNA velocity was performed using velocyto package(v.0.17.17)[21] by running "velocyto run10x" and further processed by scvelo package (version 0.2.4)[22] based on spliced and unspliced transcript reads, as previously reported.[22] The dynamical modeling (scv.tl.velocity) method was used, and only cells belonging to fibroblasts were included in the analysis. The "scv.pl.velocity_embedding_stream" function was used to project RNA velocities onto UMAP plots. All default parameters were used unless noted otherwise. The "scv.tl.paga" function was used to evaluate the relationship between different cell clusters using the partition-based graph abstraction (PAGA) analysis.[23] Trajectory analysis of CO@HAG dermal (*Dpt*$^{+}$ and *Plac8*$^{+}$) fibroblast cells was performed using the R monocle package (v.2.18.0)[24, 25] with the DDRTree method and default parameters. Raw UMI count was used as the input for Monocle2. After default normalization, the top 500 variable genes were selected by the FindVariableFeatures function, followed by reconstructing the single-cell trajectory, estimating functional states of cells, and calculating pseudotime using the reduceDimension and orderCells function. Variation from the effects of "number of expressed genes" was subtracted by setting the residualModelFormulaStr argument to "~nGene".

**Analysis of cell-cell interactions**

To identify and visualize cell-cell interactions, we employed the R package CellChat (v.1.5.0)[26] to perform cell-cell communication analysis. Briefly, we followed the official workflow((http://www.cellchat.org/)), loaded the normalized counts into CellChat, and applied standard preprocessing steps, including those using the functions "identifyOverExpressedGenes," "identifyOverExpressedInteractions," and "projectData" with a standard parameter set. We selectively used a total of 2,017 precompiled mouse ligand-receptor interactions (named "CellChatDB.mouse") as a priori network information. In each individual sample, we calculated the potential

ligand-receptor interactions among cells based on the functions "computeCommunProb," "computeCommunProbPathway," and "aggregateNet." The distance of signaling networks between untreated and CO@HAG samples and network centrality scores were calculated using the function "rankSimilarity" and "netAnalysis_computeCentrality," respectively. The function "netAnalysis_signalingRole_network" was applied to the network to determine the senders and receivers. Significant interactions associated with the TGF-β signaling pathway were extracted using the function "extractEnrichedLR". We used all standard parameters unless otherwise noted.

**Statistical analysis**

Results are presented as the mean ± standard deviation (SD) or mean ± standard error of the mean (s.e.m.). Statistical significance between the different groups were evaluated using one-way ANOVA with posthoc multiple comparisons test in IBM SPSS Statistics 26.0 software. All data were collected from at least three repeated samples(n≥3) unless otherwise noted.

**Data Availability**

Raw read sequencing files (FASTQ), as well as unfiltered read count expression matrices, were submitted to the Gene Expression Omnibus (GEO) database with accession number GSE228235.

**Code Availability**

All data were analyzed with standard programs and packages as detailed above. The scripts used in this project are available upon request to the corresponding author.

## Supplementary figures

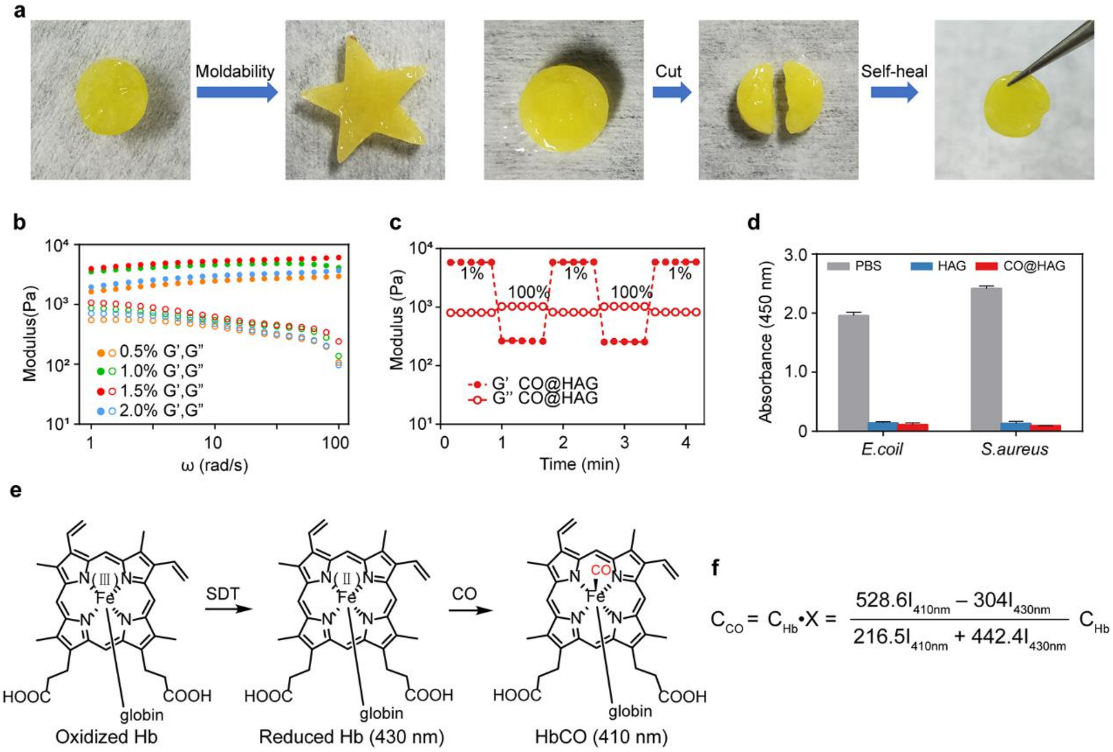


$$C_{CO} = C_{Hb} \cdot X = \frac{528.6 I_{410nm} - 304 I_{430nm}}{216.5 I_{410nm} + 442.4 I_{430nm}} C_{Hb}$$

**Figure S1.** CO@HAG formations and characterizations. (a) CO@HAG of moldable properties and self-healing properties. (b) G' and G" values of the hydrogels with 0.5%, 1.0%, 1.5% and 2.0% (w/v) of $Mn_2[CO]_{10}$ under rheological experiment with angular frequency sweep (1-100 rad/s) at fixed 0.5% strain. (c) G' and G" values of CO@HAG in the process of alternative low (1%)-high (100%)-low (1%) shear strain cycle. (d) CCK8 experiments to investigate hydrogels anti-bacterial properties. (n = 6 to 9; mean ± s.d.). (e) The mechanism of hemoglobin (Hb) method to detect the released CO gas. (f) The equation to calculate CO amount where $C_{CO}$ and $C_{Hb}$ were the released CO and the initial fed Hb concentrations, respectively, and $I_{410}$nm and $I_{430}$nm represent the UV absorbances at 410 nm and 430 nm, respectively.

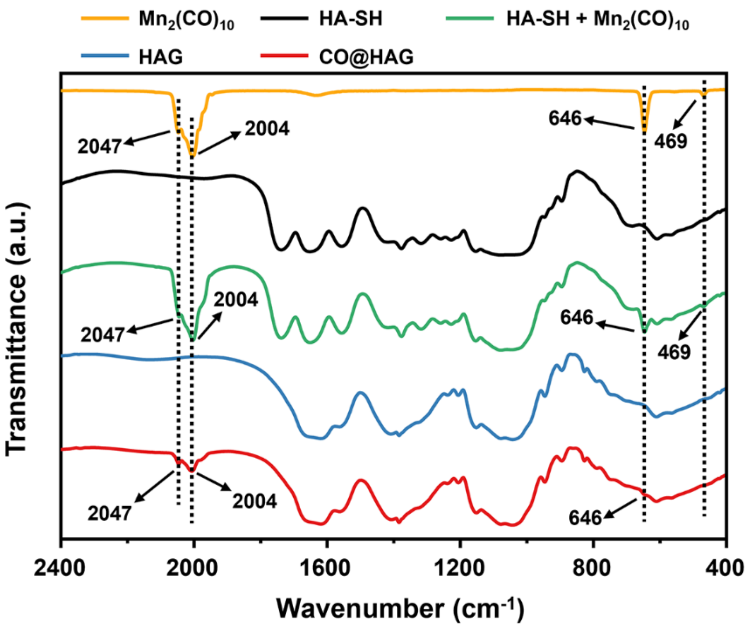


**Figure S2.** FT-IR curves for CO@HAG, HAG, HA-SH+$Mn_2[CO]_{10}$, HA-SH, $Mn_2[CO]_{10}$ samples.

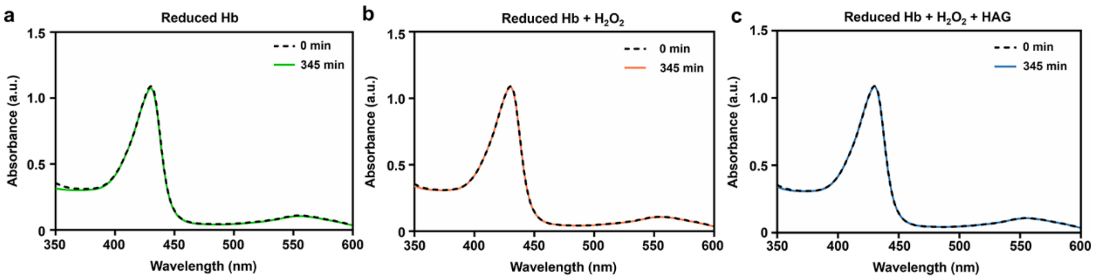


**Figure S3.** UV-vis absorbance curves of reduced Hb sample (a), reduced Hb after $H_2O_2$ addition (b), and reduced Hb + $H_2O_2$ mixture after adding HAG hydrogel (c).

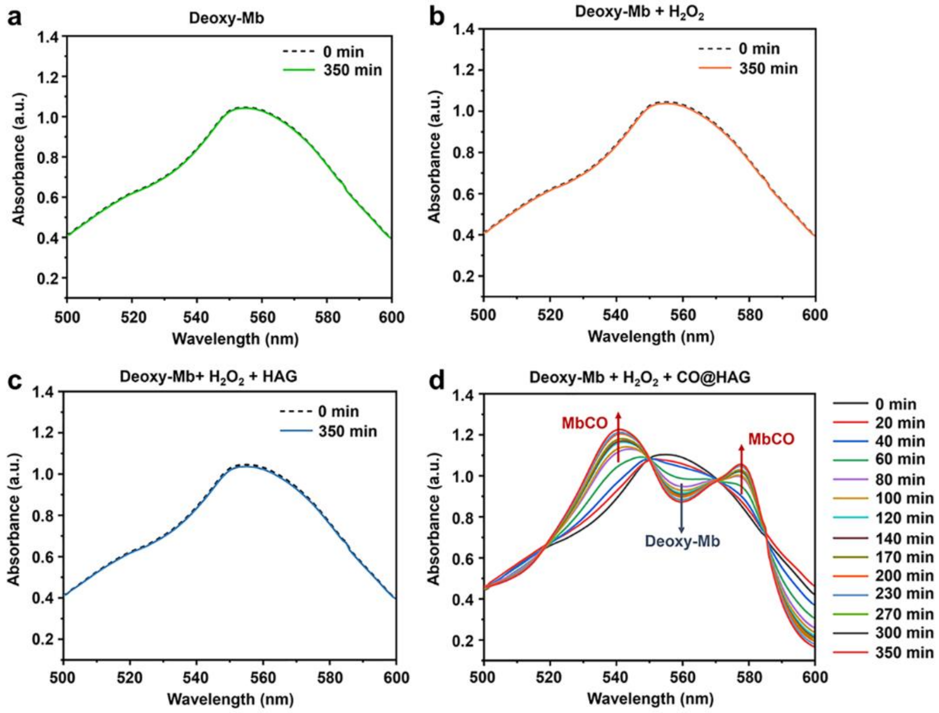


**Figure S4.** UV-vis absorbance curves of Deoxy-Mb (a), Deoxy-Mb after $H_2O_2$ addition (b), Deoxy-Mb Hb + $H_2O_2$ mixture after adding HAG hydrogel (c), Deoxy-Mb Hb+ $H_2O_2$ mixture after adding CO@HAG hydrogel(d).

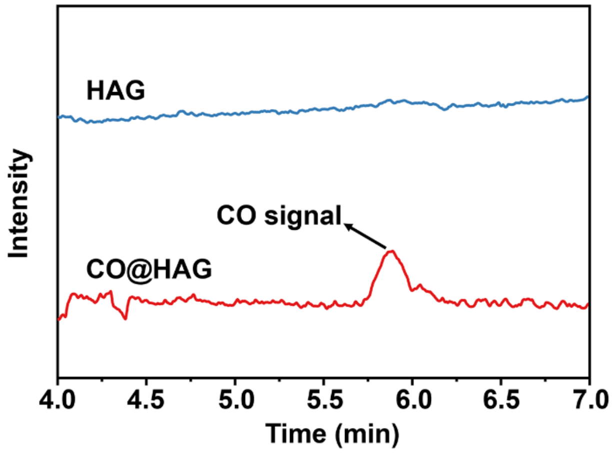


**Figure S5.** Gas chromatography analysis of CO release from CO@HAG and HAG in the presence of $H_2O_2$ (5 μM).

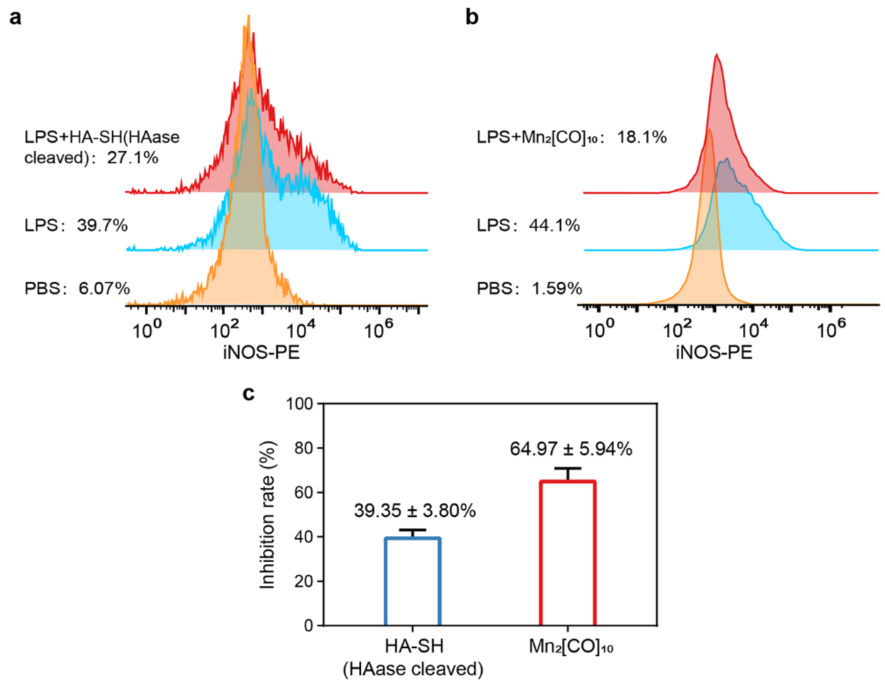


**Figure S6.** Flow cytometry analysis results. (a, b) Flow cytometry analysis of iNOS expression of LPS-induced macrophage treated by HA-SH and $Mn_2[CO]_{10}$. (c) Based on flow cytometry analysis, the value of average inhibition rate of HA-SH and $Mn_2[CO]_{10}$ against iNOS-positive macrophage (n = 3; mean ± s.e.m.).

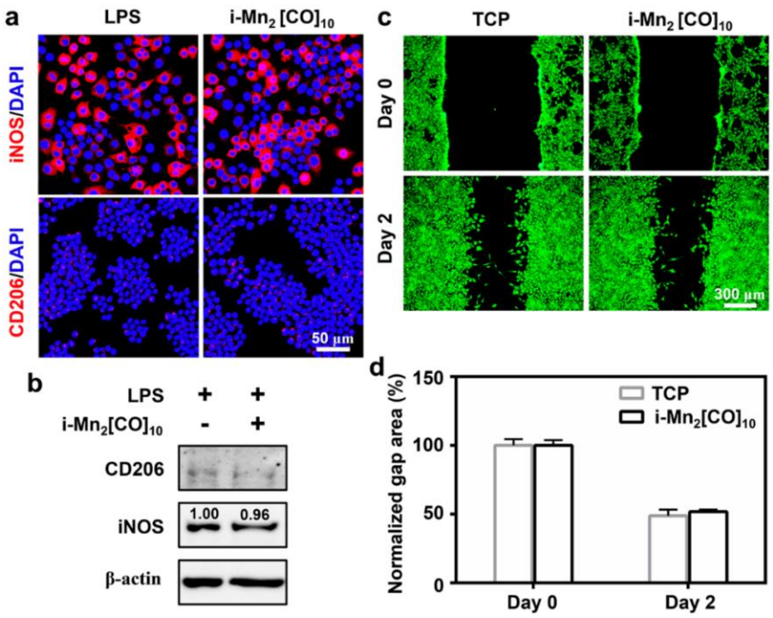


**Figure S7.** Immunofluorescence staining (a) and WB assay (b) for iNOS and CD206 in LPS- and i-$Mn_2[CO]_{10}$-treated RAW 264.7 macrophage. NIH/3T3 fibroblast of immigration assay after i-$Mn_2[CO]_{10}$ treating (c,d) (n=3; mean ± s.d.).

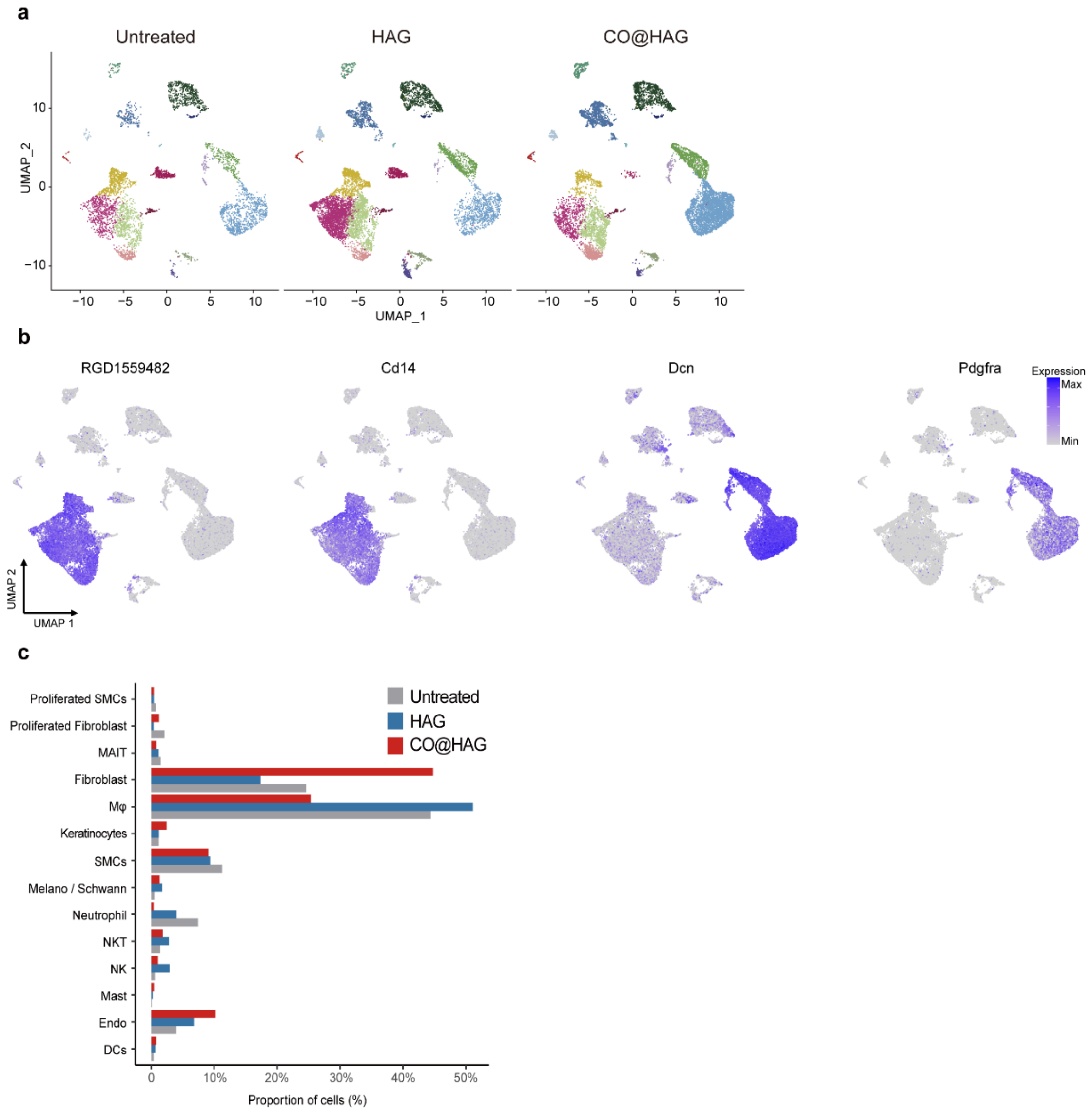


**Figure S8.** CO@HAG regulated regenerating wound of cellular heterogeneity. (a) Uniform Manifold Approximation and Projection (UMAP) plot of all cells from the untreated, HAG, and CO@HAG samples, respectively. (b) Expression of fibroblast and macrophage marker genes (*RGD1559482*, *Cd14*, *Dcn*, and *Pdgfra*) overlaid onto the UMAP plot, as shown in Fig.3h. (c) Barplots showing the proportion of different cell types in the untreated, HAG, and CO@HAG samples, respectively.

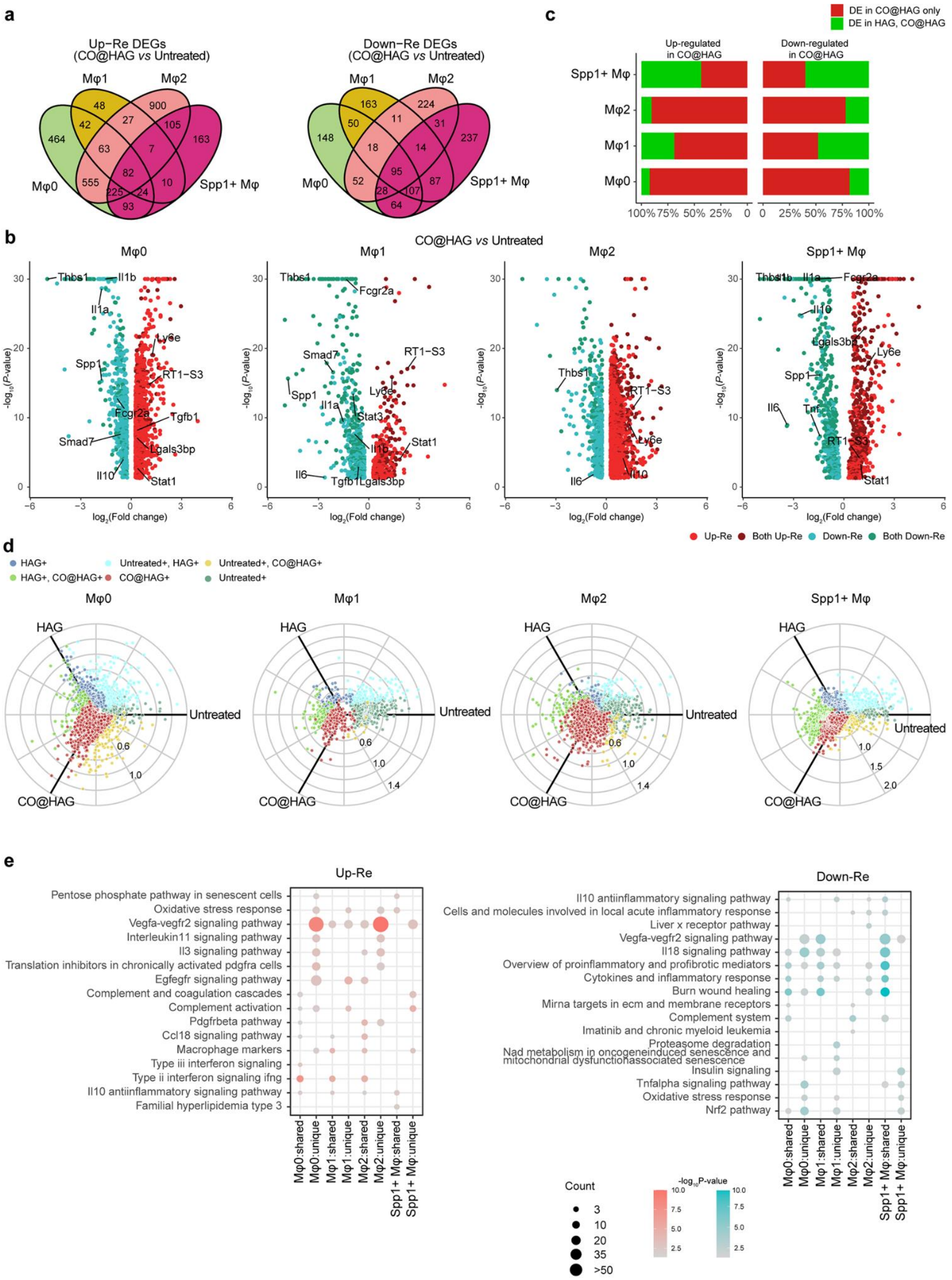


**Figure S9.** CO@HAG and HAG differentially regulated wound macrophage of inflammatory responses. (a) Venn diagrams showing overlapping up-regulated and down-regulated DEGs in the CO@HAG sample for four types of macrophages compared with untreated sample. (b) Volcano plot showing the fold change in expression and adjusted P-values (FDR) of DEGs in macrophage cells when comparing CO@HAG and untreated samples. The DEGs that are also differently expressed in

HAG samples are color-coded in different sets of colors and labeled "both" in the legend. All significant DEGs can be found in Supplemental Table S2. (c) Barplot showing the proportion of genes which were differentially expressed in both CO@HAG and HAG samples or in CO@HAG sample only. (d) Radial plot showing significant differences in gene expression between the untreated (green), HAG (blue), and CO@HAG (red) samples, and significant genes that overlap are color-coded. Scaled gene expression was internally estimated by the volcano3D package. (e) Pathway enrichment analysis of significantly DEGs where were differentially expressed in both CO@HAG and HAG samples or in CO@HAG sample only. Circle size and color represent the number of DEGs and pathway significance, respectively.

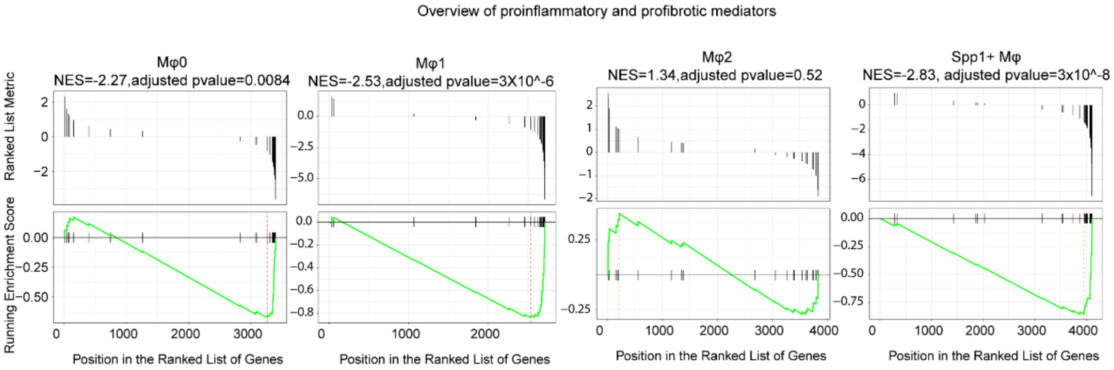


**Figure S10.** Genes related to "overview of pro-inflammatory and profibrotic mediators" were differentially expressed in different macrophage clusters after CO@HAG treatment. Plots of enrichment scores for genes with at least a 0.1 log2(Fold change) difference between untreated and CO@HAG samples in the "Overview of proinflammatory and profibrotic mediators" gene set across different sub-clusters of macrophages. Genes were sorted by log2(fold change). A higher enrichment score indicates over-representation of the genes, and a negative score indicates under-representation.

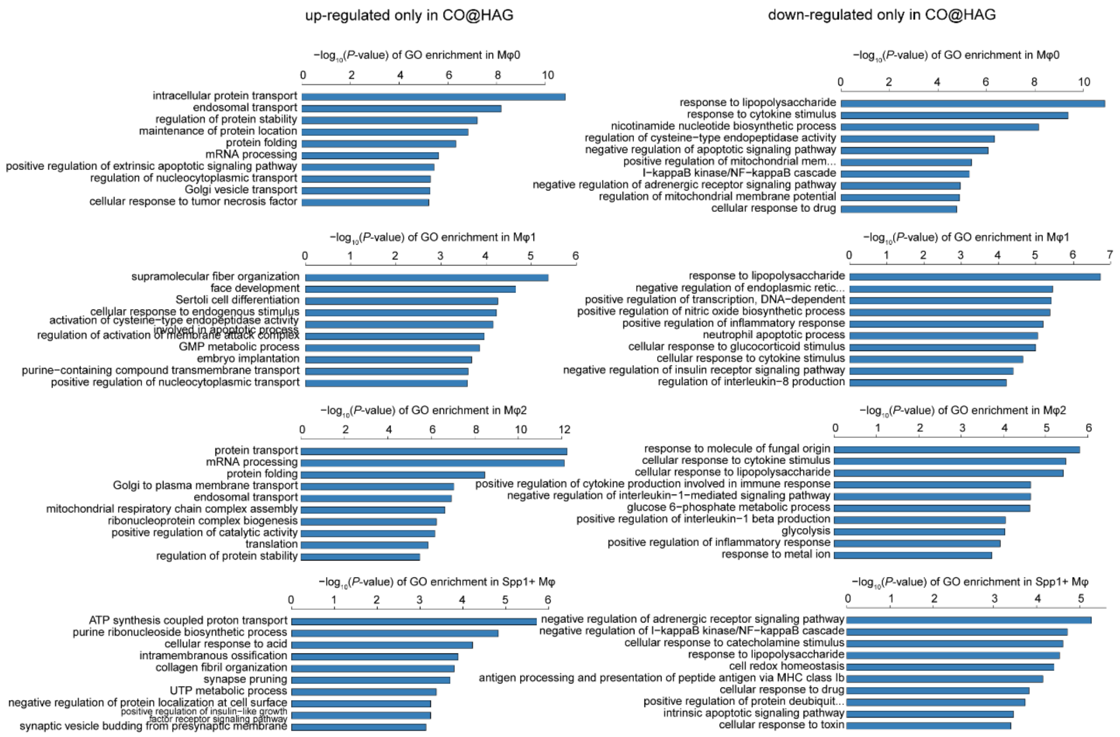


**Figure S11.** Barplot depicting the top 10 enriched biological pathway Gene Ontology (GO) terms for genes that were exclusively differentially expressed in CO@HAG samples but not in HAG samples

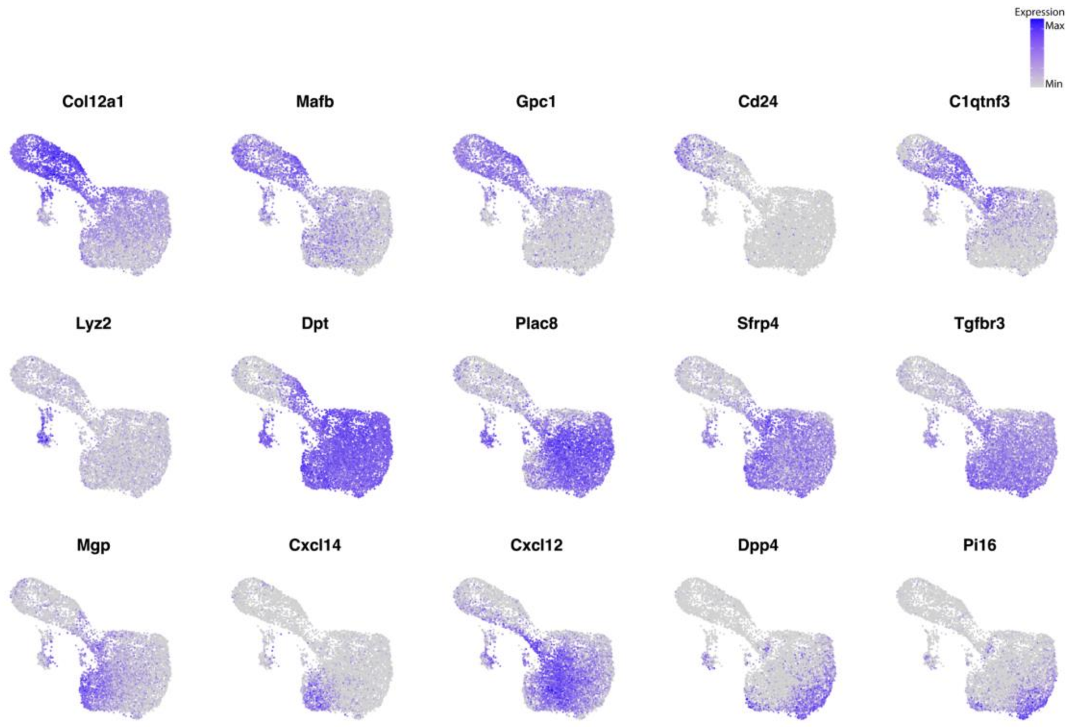


**Figure S12.** FeaturePlot showing the expression of typical marker genes for subtypes of fibroblasts in all samples.

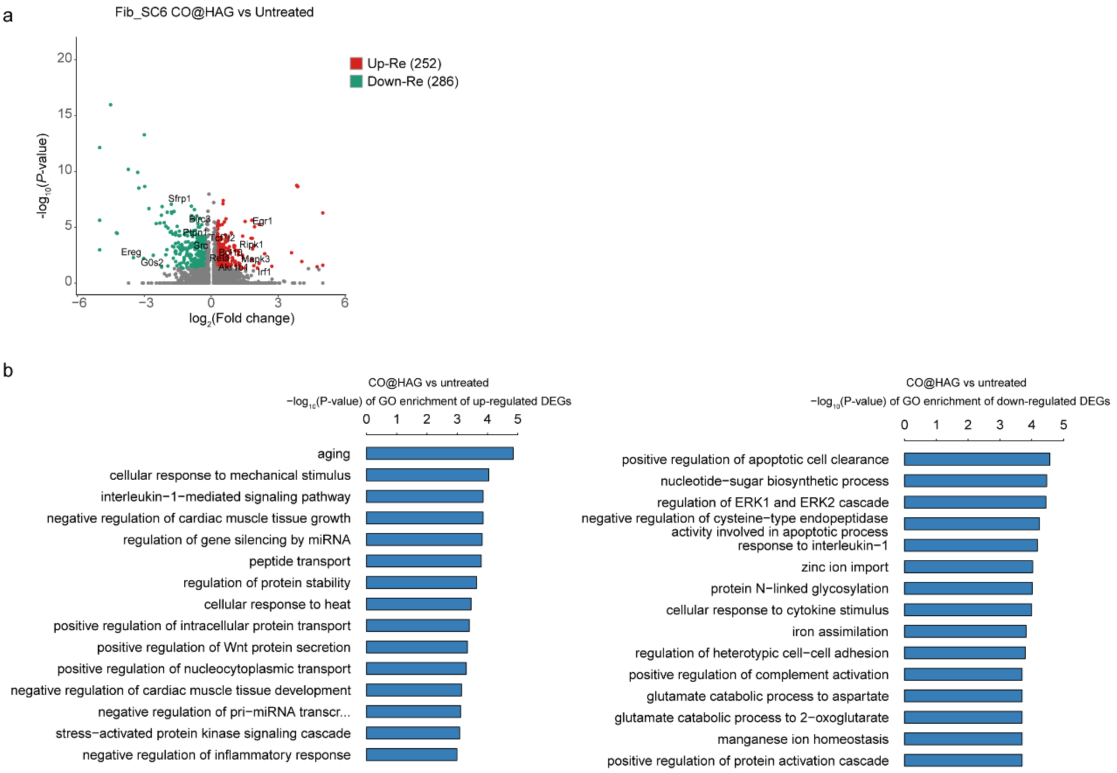


**Figure S13.** DEGs between CO@HAG and untreated in Fib_SC6 cells (a), along with the corresponding top 10 enriched biological pathway Gene Ontology (GO) terms (b).

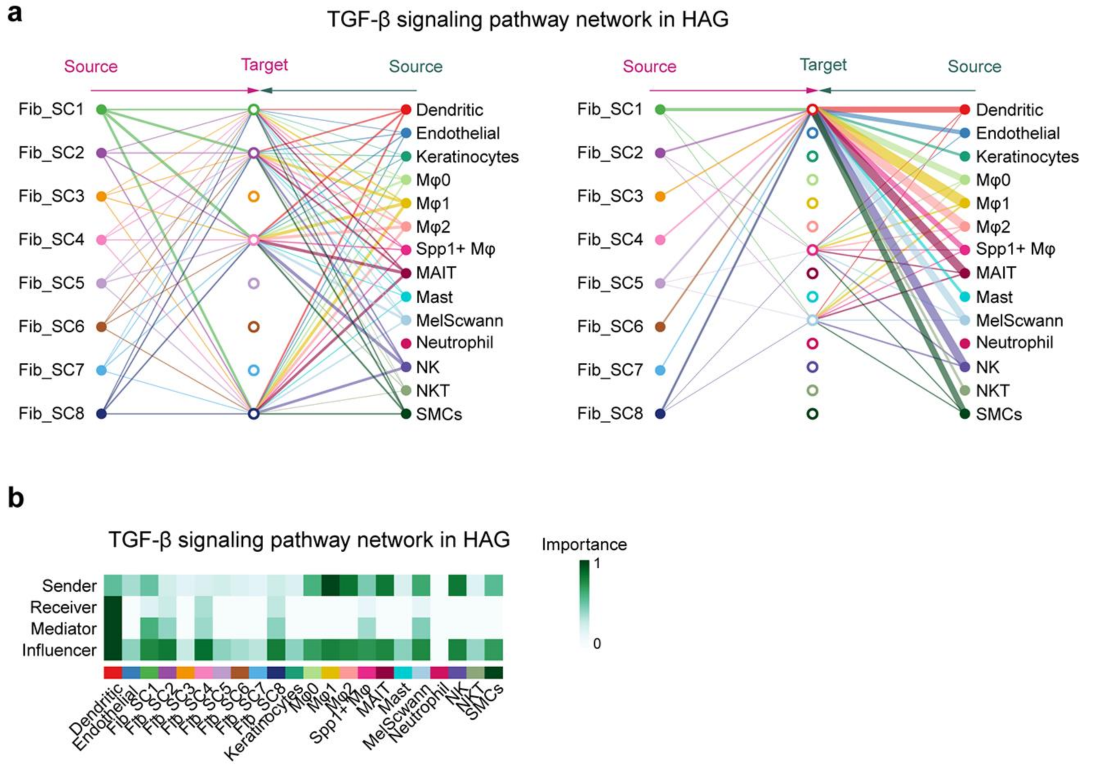


**Figure S14.** Cell-cell interactions in HAG group. (a) Hierarchical plot showing

fibroblast cell and non-fibroblast cell interactions via TGF-β signaling in HAG sample. The left and right portions show the autocrine and paracrine signaling to fibroblast cells and non-fibroblast cells, respectively. Circle sizes are proportional to the number of cells in each cell group, and edge width represents the communication probability. (b)The computed network centrality measures of TGF-β signaling.

## References


(1) Ossipov, D. A.; Yang, X.; Varghese, O.; Kootala, S.; Hilborn, J. Modular approach to functional hyaluronic acid hydrogels using orthogonal chemical reactions. *Chem. Commun.* **2010**, *46*, 8368-8370.

(2) Chernousova, S.; Epple, M. Silver as antibacterial agent: ion, nanoparticle, and metal. *Angew. Chem. Int. Ed.* **2013**, *52*, 1636-1653.

(3) Atiyeh, B. S.; Costagliola, M.; Hayek, S. N.; Dibo, S. A. Effect of silver on burn wound infection control and healing: Review of the literature. *Burns* **2007**, *33*, 139-148.

(4) Shi, L.; Zhao, Y.; Xie, Q.; Fan, C.; Hilborn, J.; Dai, J.; Ossipov, D. A. Moldable Hyaluronan Hydrogel Enabled by Dynamic Metal-Bisphosphonate Coordination Chemistry for Wound Healing. *Adv. Healthcare Mater.* **2018**, *7*, 1700973.

(5) He, Q.; Kiesewetter, D. O.; Qu, Y.; Fu, X.; Fan, J.; Huang, P.; Liu, Y.; Zhu, G.; Liu, Y.; Qian, Z.; et al. NIR-Responsive On-Demand Release of CO from Metal Carbonyl-Caged Graphene Oxide Nanomedicine. *Adv. Mater.* **2015**, *27*, 6741-6746.

(6) Zheng, G. X. Y.; Terry, J. M.; Belgrader, P.; Ryvkin, P.; Bent, Z. W.; Wilson, R.; Ziraldo, S. B.; Wheeler, T. D.; McDermott, G. P.; Zhu, J.; et al. Massively parallel digital transcriptional profiling of single cells. *Nat. Commun.* **2017**, *8*, 14049.

(7) Cunningham, F.; Allen, J. E.; Allen, J.; Alvarez-Jarreta, J.; Amode, M. R.; Armean, I. M.; Austine-Orimoloye, O.; Azov, A. G.; Barnes, I.; Bennett, R.; et al. Ensembl 2022. *Nucleic Acids Res.* **2022**, *50*, D988-D995.

(8) McGinnis, C. S.; Murrow, L. M.; Gartner, Z. J. DoubletFinder: Doublet Detection in Single-Cell RNA Sequencing Data Using Artificial Nearest Neighbors. *Cell Syst.* **2019**, *8*, 329-337.

(9) Lun, A. T. L.; McCarthy, D. J.; Marioni, J. C. A step-by-step workflow for low-level analysis of single-cell RNA-seq data with Bioconductor. *F1000Research https://doi.org/10.12688/f1000research.9501.2* **2016**, *5*, 2122.

(10) Haghverdi, L.; Lun, A. T. L.; Morgan, M. D.; Marioni, J. C. Batch effects in single-cell RNA-sequencing data are corrected by matching mutual nearest neighbors. *Nat. Biotechnol.* **2018**, *36*, 421-427.

(11) Stuart, T.; Butler, A.; Hoffman, P.; Hafemeister, C.; Papalexi, E.; Mauck III, W. M.; Hao, Y.; Stoeckius, M.; Smibert, P.; Satija, R. Comprehensive Integration of Single-Cell Data. *Cell* **2019**, *177*, 1888-1902.

(12) Aran, D.; Looney, A. P.; Liu, L.; Wu, E.; Fong, V.; Hsu, A.; Chak, S.; Naikawadi,

R. P.; Wolters, P. J.; Abate, A. R.; et al. Reference-based analysis of lung single-cell sequencing reveals a transitional profibrotic macrophage. *Nat. Immunol.* **2019**, *20*, 163-172.
(13) Anders, S.; Huber, W. Differential expression of RNA-Seq data at the gene level–the DESeq package (EMBL, 2013). **2013**.
(14) Finak, G.; McDavid, A.; Yajima, M.; Deng, J.; Gersuk, V.; Shalek, A. K.; Slichter, C. K.; Miller, H. W.; McElrath, M. J.; Prlic, M.; et al. MAST: a flexible statistical framework for assessing transcriptional changes and characterizing heterogeneity in single-cell RNA sequencing data. *Genome Biol.* **2015**, *16*, 278.
(15) Goldmann, K.; Lewis, M. volcano3D: 3D volcano plots and polar plots for Three-Class data. https://github.com/KatrionaGoldmann/volcano3D. **2020**.
(16) Alexa, A.; Rahnenfuhrer, J. topGO: Enrichment Analysis for Gene Ontology. R package version 2.42.0. **2020**.
(17) Yu, G.; Wang, L.-G.; Han, Y.; He, Q.-Y. clusterProfiler: an R Package for Comparing Biological Themes Among Gene Clusters. *OMICS* **2012**, *16*, 284-287.
(18) Subramanian, A.; Tamayo, P.; Mootha, V. K.; Mukherjee, S.; Ebert, B. L.; Gillette, M. A.; Paulovich, A.; Pomeroy, S. L.; Golub, T. R.; Lander, E. S.;et al. Gene set enrichment analysis: a knowledge-based approach for interpreting genome-wide expression profiles. *Proc. Natl. Acad. Sci. U. S. A.* **2005**, *102*, 15545-15550.
(19) Liberzon, A.; Birger, C.; Thorvaldsdóttir, H.; Ghandi, M.; Mesirov, J. P.; Tamayo, P. The Molecular Signatures Database (MSigDB) hallmark gene set collection. *Cell Syst.* **2015**, *1*, 417-425.
(20) Liberzon, A.; Subramanian, A.; Pinchback, R.; Thorvaldsdóttir, H.; Tamayo, P.; Mesirov, J. P. Molecular signatures database (MSigDB) 3.0. *Bioinformatic* **2011**, *27*, 1739-1740.
(21) La Manno, G.; Soldatov, R.; Zeisel, A.; Braun, E.; Hochgerner, H.; Petukhov, V.; Lidschreiber, K.; Kastriti, M. E.; Lönnerberg, P.; Furlan, A.; et al. RNA velocity of single cells. *Nature* **2018**, *560*, 494-498.
(22) Bergen, V.; Lange, M.; Peidli, S.; Wolf, F. A.; Theis, F. J. Generalizing RNA velocity to transient cell states through dynamical modeling. *Nat. Biotechnol.* **2020**, *38*, 1408-1414.
(23) Wolf, F. A.; Hamey, F. K.; Plass, M.; Solana, J.; Dahlin, J. S.; Göttgens, B.; Rajewsky, N.; Simon, L.; Theis, F. J. PAGA: graph abstraction reconciles clustering with trajectory inference through a topology preserving map of single cells. *Genome Biol.* **2019**, *20*, 59.
(24) Qiu, X.; Hill, A.; Packer, J.; Lin, D.; Ma, Y.-A.; Trapnell, C. Single-cell mRNA quantification and differential analysis with Census. *Nat. Methods* **2017**, *14*, 309-315.
(25) Qiu, X.; Mao, Q.; Tang, Y.; Wang, L.; Chawla, R.; Pliner, H. A.; Trapnell, C. Reversed graph embedding resolves complex single-cell trajectories. *Nat. Methods* **2017**, *14*, 979-982.
(26) Jin, S.; Guerrero-Juarez, C. F.; Zhang, L.; Chang, I.; Ramos, R.; Kuan, C.-H.; Myung, P.; Plikus, M. V.; Nie, Q. Inference and analysis of cell-cell communication using CellChat. *Nat. Commun.* **2021**, *12*, 1088.
